# Processing-induced detoxification of toxic Traditional Chinese Medicines: a systematic review of attenuation mechanisms at physical, chemical, and *in vivo* levels

**DOI:** 10.1186/s13020-026-01437-6

**Published:** 2026-06-10

**Authors:** Weiye Zhang, Zhubin Zhang, Ziwen Bian, Qi Zuo, Xue Quan, Jiahao Gong, Bingling Ju, Liang Feng, Xiaobin Jia, Bing Yang

**Affiliations:** School of Traditional Chinese Pharmacy, State Key Laboratory of Natural Medicines, China Pharmaceutical University, Nanjing, 211198 People’s Republic of China

**Keywords:** Toxic Traditional Chinese Medicines (TCMs), Processing-induced detoxification, Physical detoxification, Chemical transformation, Modulation of in vivo processes, Natural source, Common patterns

## Abstract

The “toxicity” of Traditional Chinese Medicines (TCMs) carries a dual connotation, referring both to the potent nature of certain medicinal substances and a critical constraint on their safe clinical application. Processing is the core approach to achieving the goal of “attenuating toxicity while preserving efficacy” for toxic TCMs. A systematic elucidation of the detoxification mechanisms of processing is essential for inheriting traditional processing theories and ensuring clinical medication safety. Current research predominantly focuses on individual toxic TCMs, specific toxic constituents, or single processing methods, a fragmented paradigm that hinders a comprehensive understanding of the complex detoxification mechanisms and the extraction of universal principles. Therefore, this paper systematically reviews the toxic TCMs listed in the *Chinese Pharmacopoeia* (2025 Edition), summarizing their natural sources, types of toxic constituents, and traditional processing methods for toxicity reduction. Furthermore, by integrating existing research evidence, the detoxification mechanisms of processing are categorized into three interrelated yet distinct levels: physical detoxification (including the physical removal of toxic constituents and changes in the physical properties of decoction pieces), chemical transformation of toxic constituents, and modulation of in vivo processes. At the chemical transformation level, common patterns within the same family or genus are explored based on natural sources. At the physical level, the focus is on universal processing methods across different families and genera; at the in vivo level, the focus is on common regulatory pathways across families and genera. This review aims to integrate fragmented research findings, reveal potential common patterns underlying processing‑induced detoxification, and propose an integrated research strategy combining “physical detoxification – chemical transformation of toxic constituents – modulation of in vivo processes.” It thereby provides a theoretical basis for a deeper understanding of the scientific principles of processing‑induced detoxification and for guiding the optimization and innovation of processing technologies.

**Graphical Abstract:**

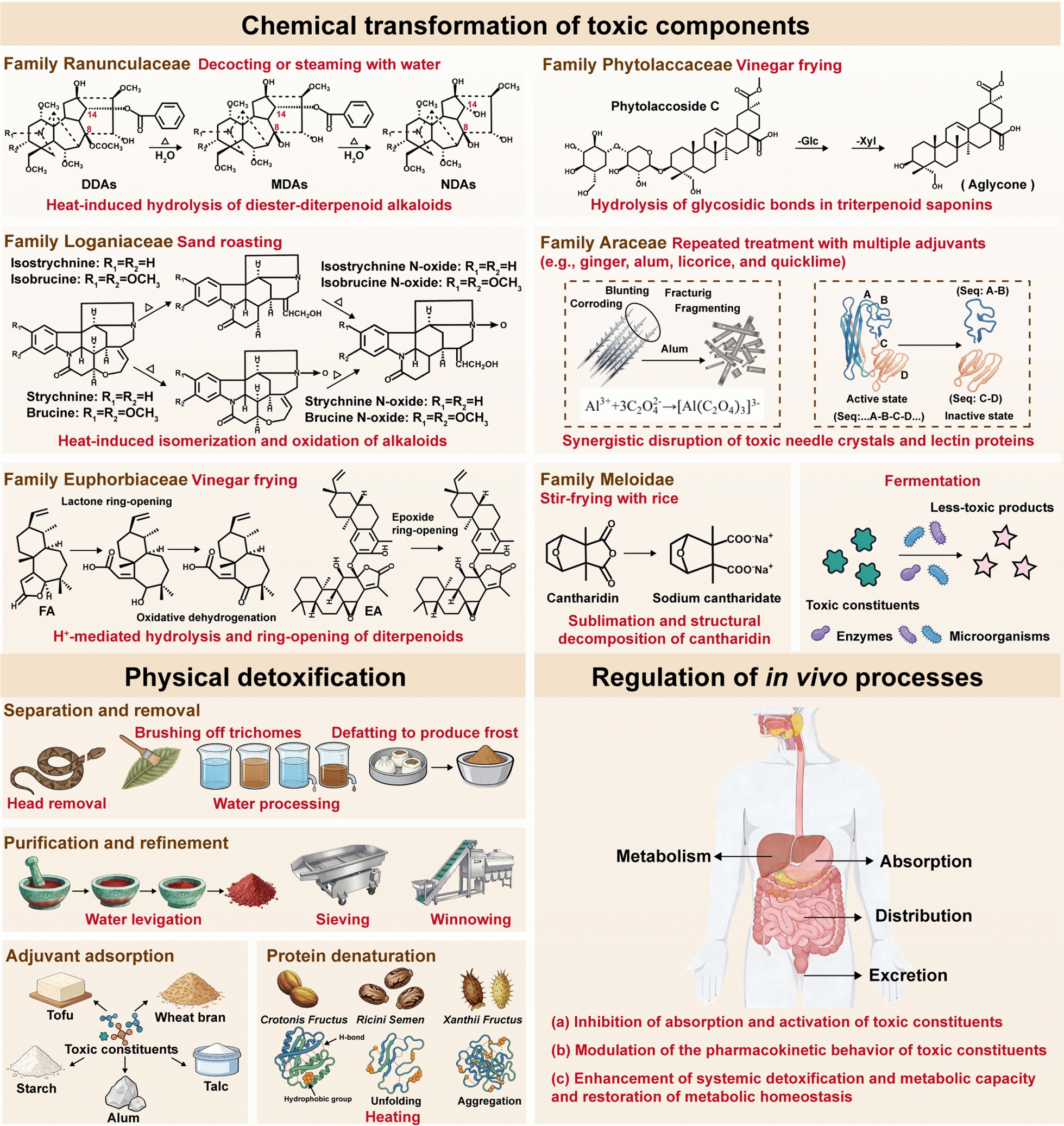

## Background

The “toxicity” of Traditional Chinese Medicines (TCMs) is central to the theory of medicinal properties and carries a dual connotation [1]. Within the theoretical framework of Traditional Chinese Medicine (TCM), “toxicity” refers to the potent and drastic nature of certain medicinal substances, characterized by their robust therapeutic capacity to rectify physiological imbalances through the principle of “using toxicity to counteract toxicity,” thereby achieving therapeutic outcomes [2]. From a modern scientific perspective, however, this same potency can cause substantial harm to the organism when the dosage exceeds the therapeutic range or when the clinical application is inappropriate [3]. Such harm can manifest as stimulation or damage to the central nervous system, digestive system, and reproductive system, impairment of hepatorenal function, as well as corrosive effects on local tissues such as the skin, mucous membranes, and muscles [4–8]. It is precisely this intricate interplay between toxicity and therapeutic efficacy that imposes significant clinical constraints on the application of potent yet toxic TCMs, as exemplified by *Aconiti Radix* (Chuanwu), *Aconiti Kusnezoffii Radix* (Caowu), *Aconiti Radix Lateralis Praeparata* (Fuzi), *Kansui Radix* (Gansui), and *Pinelliae Rhizoma* (Banxia) [9–11]. Therefore, the fundamental objective of processing toxic TCMs is to reduce or control their inherent toxicity and mitigate potential adverse reactions through appropriate methods, while optimally preserving their therapeutic efficacy [12, 13]. This ultimately achieves the goal of “attenuating toxicity while preserving efficacy,” thereby facilitating the safe and effective clinical application of these toxic TCMs in modern therapeutic practice.

To achieve the goal of “attenuating toxicity while preserving efficacy,” a systematic investigation into the detoxification mechanisms underlying processing methods is essential [14]. This endeavor not only serves as a foundation for elucidating the scientific rationale of traditional processing techniques but also constitutes a critical step to ensuring clinical medication safety. Although significant progress has been made, current research predominantly focuses on individual toxic TCMs, specific toxic constituents, or single processing methods. The limitations of this fragmented research paradigm are becoming increasingly evident: the lack of systematic integration and comparison among numerous isolated studies hampers a comprehensive understanding of the complex mechanisms underlying processing-induced detoxification and impedes the extraction of universal principles for toxicity attenuation. In the process of systematically synthesizing the existing literature, it becomes evident that the detoxification mechanisms of processing are inherently multidimensional, involving three independent yet interrelated levels: physical detoxification (including physical removal of toxic constituents and changes in the physical properties of decoction pieces), chemical transformation of toxic constituents, and modulation of in vivo processes. However, there is currently a lack of literature that systematically integrates these three levels. Furthermore, toxic TCMs derived from the same natural source category (e.g., specific plant families or genera) often contain identical or similar types of toxic constituents and are commonly processed using analogous strategies, suggesting the potential existence of significant family- or genus-level commonalities at the chemical transformation level—commonalities closely associated with their conserved secondary metabolite profiles [15, 16]. In contrast, detoxification principles at the physical level tend to manifest as cross-family or cross-genus universal measures (e.g., water levigation, adjuvant adsorption, removal of toxic parts) rather than family- or genus-specific rules. By comparison, regulatory mechanisms at the in vivo level are influenced by multiple factors. The absorption, distribution, metabolism, and excretion processes of toxic constituents in the body are highly complex, involving the coordinated regulation of multiple organs and systems, including gastrointestinal tract, liver, kidneys, and gut microbiota. Alteration in any of these links may change the overall disposition characteristics of toxic constituents. Therefore, identifying family- or genus-specific regularities at this level remains challenging at present.

On the basis of the above findings, this review focuses on TCMs labeled as toxic in the *Chinese Pharmacopoeia* (2025 Edition). Their natural sources, types of toxic constituents, and traditional processing methods for toxicity attenuation are systematically summarized. By integrating existing fragmented studies on individual toxic TCMs, the evidence and mechanisms of processing-induced detoxification are classified into three levels: physical detoxification, chemical transformation of toxic constituents, and modulation of in vivo processes. Subsequently, at the chemical transformation level, an in-depth inductive analysis is conducted based on natural sources (family/genus) to explore common patterns in constituent transformation pathways among different toxic TCMs within the same family or genus. At the physical level, the focus is on common processing methods across different families and genera; at the in vivo level, the focus is on common regulatory pathways across families and genera. Ultimately, this review aims to integrate fragmented research findings, reveal potential common patterns underlying processing-induced detoxification, and propose an integrated research strategy combining “physical detoxification – chemical transformation of toxic constituents – modulation of in vivo processes,” thereby providing a theoretical basis for a deeper understanding of the scientific principles underlying processing-induced detoxification and for guiding the optimization and innovation of processing technologies.

## Methods

In this review, a systematic and comprehensive literature search was performed, covering publications up to May 8, 2026, from authoritative academic databases including PubMed (https://pubmed.ncbi.nlm.nih.gov), Web of Science (https://www.webofscience.com), and the China National Knowledge Infrastructure (CNKI, https://www.cnki.net/). The search strategy consisted of two parts. First, for all toxic TCMs included in the *Chinese Pharmacopoeia* (2025 edition), their Chinese names, common English names, and Latin scientific names were used as search terms in combination with keywords such as “toxicity”, “processing”, “detoxification”, and “toxicity reduction”. Second, a supplementary search was performed using the following keywords, either individually or in combination: “toxic Traditional Chinese Medicines,” “processing,” “detoxification,” “toxicity reduction,” “plant-derived drugs,” “animal-derived drugs,” “mineral-derived drugs,” “toxic constituents,” “alkaloids,” “terpenoids,” “glycosides,” and “toxic proteins”. The retrieved literature records were imported into the reference management software EndNote. After removing duplicates, a three‑stage screening process was performed by reading titles, abstracts, and full texts. The inclusion criteria were as follows: (a) the research subjects were TCMs clearly marked in Part I of the *Chinese Pharmacopoeia* (2025 edition) as possessing “major toxicity”, “toxicity”, or “minor toxicity”; (b) the study explicitly involved the processing of the toxic TCMs and focused on their toxicity‑reducing effects; (c) the study types included analytical chemistry studies revealing changes in chemical constituents before and after processing, in vivo or in vitro pharmacological/toxicological experimental studies evaluating toxicity changes before and after processing, studies exploring the molecular mechanisms of toxicity reduction, and relevant systematic reviews; (d) the publication language was limited to Chinese or English; and (e) the full text had to be accessible. The exclusion criteria were as follows: (a) studies published only as abstracts, conference papers, comments, or letters; (b) duplicate publications or studies with incomplete data; and (c) studies that only reported toxic phenomena without addressing the specific processing‑induced toxicity‑reduction mechanisms. Any disagreements were resolved through discussion or by arbitration of a third researcher. The entire screening process adhered strictly to systematic and rigorous principles.

### Natural source, chemical constituents, and toxicological mechanisms of toxic TCMs

The *Chinese Pharmacopoeia* (2025 Edition), as the supreme statutory standard for drugs in China, endows the crude drugs and decoction pieces it includes with the highest authority and broad representativeness. Through systematic screening, a total of 83 toxic TCMs explicitly labeled as “major toxicity”, “toxic”, or “minor toxicity” in Part I of this edition have been identified, comprising 70 plant-derived, 8 animal-derived, and 5 mineral-derived TCMs [17]. On this basis, this paper systematically reviews the natural sources, major types of toxic constituents, and traditional processing methods for toxicity reduction of the above-mentioned toxic TCMs, aiming to provide a foundation for investigating the detoxification mechanisms of processing. During this systematic review, it was found that plant-derived toxic TCMs involve 35 families. Among them, toxic TCMs from certain families such as Ranunculaceae, Loganiaceae, Euphorbiaceae, Araceae, and Phytolaccaceae have had their standard detoxification processing methods recorded in the *Chinese Pharmacopoeia*, and certain research achievements in chemistry and toxicology have been made. Notably, certain toxic TCMs within the same family or genus often exhibit significant commonalities in their chemical constituents and toxicity characteristics. This may be closely related to their conserved secondary metabolite profiles formed over long-term evolution—these metabolites form the shared material basis for both therapeutic efficacy and toxicity, providing critical phytochemical clues for investigating how processing specifically transforms or degrades certain types of toxic constituents. Among animal-derived toxic TCMs, except for cantharidin in *Mylabris* (Banmao), which is a small organic molecule [18, 19], the toxic constituents are primarily proteins or peptides [20]. This common feature determines the consistency of their detoxification strategies (e.g., removal of toxic parts, heat-induced protein denaturation). The toxicity of mineral-derived TCMs fundamentally depends on their inherent physicochemical properties, including chemical composition, crystal structure, solubility, and elemental valence states. Based on this, targeted processing methods such as water levigation can be selected to achieve detoxification. The principal toxic constituents, toxicological mechanisms, and detoxification processing methods of representative toxic TCMs from different natural sources are summarized in Table 1.

**Table 1:** The principal toxic constituents, toxicological mechanisms, and detoxification processing methods of representative toxic TCMs from different natural sources

Natural source	Taxonomic classification	Representative toxic TCMs	Principal toxic constituents	Toxicological manifestations	Toxic mechanisms	Processing methods	Refs
Plant origin	Family Ranunculaceae, Genus *Aconitum*	*Aconiti Radix Lateralis Praeparata* (Fuzi); *Aconiti Radix* (Chuanwu); *Aconiti Kusnezoffii Radix* (Caowu)	Diester-diterpenoid alkaloids (DDAs, e.g., aconitine, mesaconitine, and hypaconitine)	Cardiotoxicity; Neurotoxicity; Hepatotoxicity; Reproductive toxicity	Cardiotoxicity/neurotoxicity: Persistent activation of voltage‑gated Na^+^ channels leading to disruption of electrical signal transduction in the myocardium and nervous system; Hepatotoxicity: Characterized by the disruption of metabolic and bile acid homeostasis, coupled with the induction of liver injury through the activation of oxidative stress and inflammatory pathways; Reproductive toxicity: Interference with the genetic programming of embryonic development and induction of DNA damage	Decocting or steaming with water, or co‑processing with adjuvants (e.g., Danba solution, licorice, and black soybean)	[23–35]
Plant origin	Family Loganiaceae, Genus *Strychnos*	*Strychni Semen* (Maqianzi)	Indole alkaloids (e.g., strychnine and brucine)	Central neurotoxicity; Nephrotoxicity	Central neurotoxicity: Potent competitive antagonism of glycine receptors; Nephrotoxicity: Accumulation of alkaloids in renal cells, leading to disruption of tyrosine metabolism	Sand roasting	[38–46]
Plant origin	Family Euphorbiaceae, Genus *Euphorbia*	*Kansui Radix* (Gansui); *Euphorbiae Pekinensis Radix* (Jingdaji); *Euphorbiae Ebracteolatae Radix* (Langdu)	Diterpenoids	Acute gastrointestinal toxicity; Secondary systemic toxicity	Direct cytotoxicity disrupting membrane structures; Induction of oxidative stress and triggering of a robust inflammatory cascade	Vinegar frying	[48–56]
Plant origin	Family Euphorbiaceae, Genus *Euphorbia*	*Euphorbiae Semen* (Qianjinzi)	Diterpenoid esters (e.g., euphorbiasteroid)	Acute gastrointestinal toxicity; Secondary systemic toxicity	Fatty oil‑mediated intestinal lubrication and exacerbation of diarrhea; Direct cytotoxicity; Induction of oxidative stress and triggering of a robust inflammatory cascade	Defatting to produce frost	[57, 58]
Plant origin	Family Euphorbiaceae, Genus *Croton*	*Crotonis Fructus* (Badou)	Croton oil (e.g., phorbol 12-myristate 13-acetate); Crotin (e.g., croton protein I and II)	Acute gastrointestinal toxicity; Secondary systemic toxicity	Phorbol 12-myristate 13-acetate: Activation of PKC triggering robust inflammatory signaling; Crotin: Direct cytotoxicity and hemolytic activity	Defatting to produce frost	[59–62]
Plant origin	Family Euphorbiaceae, Genus *Ricinus*	*Ricini Semen* (Bimazi)	Ricin; Ricinine	Acute gastrointestinal toxicity; Secondary systemic toxicity	Inhibition of protein synthesis; Induction of intracellular internalization; Direct mucosal damage	Stir-frying	[63–65]
Plant origin	Family Phytolaccaceae, Genus *Phytolacca*	*Phytolaccae Radix* (Shanglu)	Triterpenoid saponin (e.g., phytolaccoside A, B, C, and H)	Acute gastrointestinal toxicity; Neurotoxicity; Hepatotoxicity	Acute gastrointestinal toxicity: Direct physical irritation to the gastrointestinal mucosa, along with activation of macrophages and release of pro-inflammatory cytokines; Neurotoxicity: Phytolaccoside B inhibits acetylcholinesterase activity, disrupting cholinergic signaling and thereby impairing central nervous system development in zebrafish larvae; Hepatotoxicity: Phytolaccoside A induces oxidative stress and energy metabolism disorders, subsequently activating the P53‑dependent apoptosis pathway and ferroptosis	Vinegar frying	[69–73]
Plant origin	Family Araceae	*Pinelliae Rhizoma* (Banxia); *Arisaematis Rhizoma* (Tiannanxing); *Typhonii Rhizoma* (Baifuzi)	Toxic needle crystals; Lectin proteins	Irritant‑induced inflammatory response; Maternal and developmental toxicity	Mechanical irritation from needle crystals and chemical damage caused by lectin proteins	Repeated treatment with multiple adjuvants (e.g., ginger, alum, licorice, and quicklime)	[75–83]
Animal origin	Family Bufonidae	*Bufonis Venenum* (Chansu)	Bufadienolides (e.g., bufalin, cinobufotalin, cinobufagin and resibufogenin)	Cardiotoxicity	Induction of intracellular calcium overload via inhibition of Na^+^/K^+^-ATPase activity	Maceration with white liquor	[102–104]
Animal origin	Family Haemopidae; Family Hirudinidae	*Hirudo* (Shuizhi)	H irudin	Systemic bleeding tendency	Thrombin inhibition	Stir‑frying with heated talcum powder	[99, 105]
Animal origin	Family Meloidae	*Mylabris* (Banmao); *Lytta Caraganae* (Qingniangzi)	Cantharidin	Irritant‑induced inflammatory response; Nephrotoxicity; Hepatotoxicity	Irritant-induced inflammatory response: Induction of inflammatory mediator release; Nephrotoxicity: Inhibition of PP2A activity triggers widespread metabolic and signal transduction disturbances, subsequently inducing oxidative stress, endoplasmic reticulum stress, and aberrant activation of signaling pathways; Hepatotoxicity: Metabolic disturbances lead to endoplasmic reticulum stress and mitochondrial dysfunction, ultimately triggering autophagy, apoptosis, and necrosis in hepatocytes	Stir‑frying with rice	[106–117]
Mineral origin	/	*Cinnabaris* (Zhusha)	Soluble mercury salts	Nephrotoxicity	High affinity of Hg^2+^ for -SH groups, inhibition of the renal detoxification system, and overactivation of the innate immune inflammatory pathway	Water levigation	[118–123]
Mineral origin	/	*Realgar* (Xionghuang)	Trivalent arsenic (As_2_O_3_)	Systemic multi-organ toxicity	High affinity of As^3+^ for -SH groups, oxidative stress, and cellular damage	Water levigation	[124–126]

### Plant-derived toxic TCMs

#### Family Ranunculaceae

**Fig. 1:**
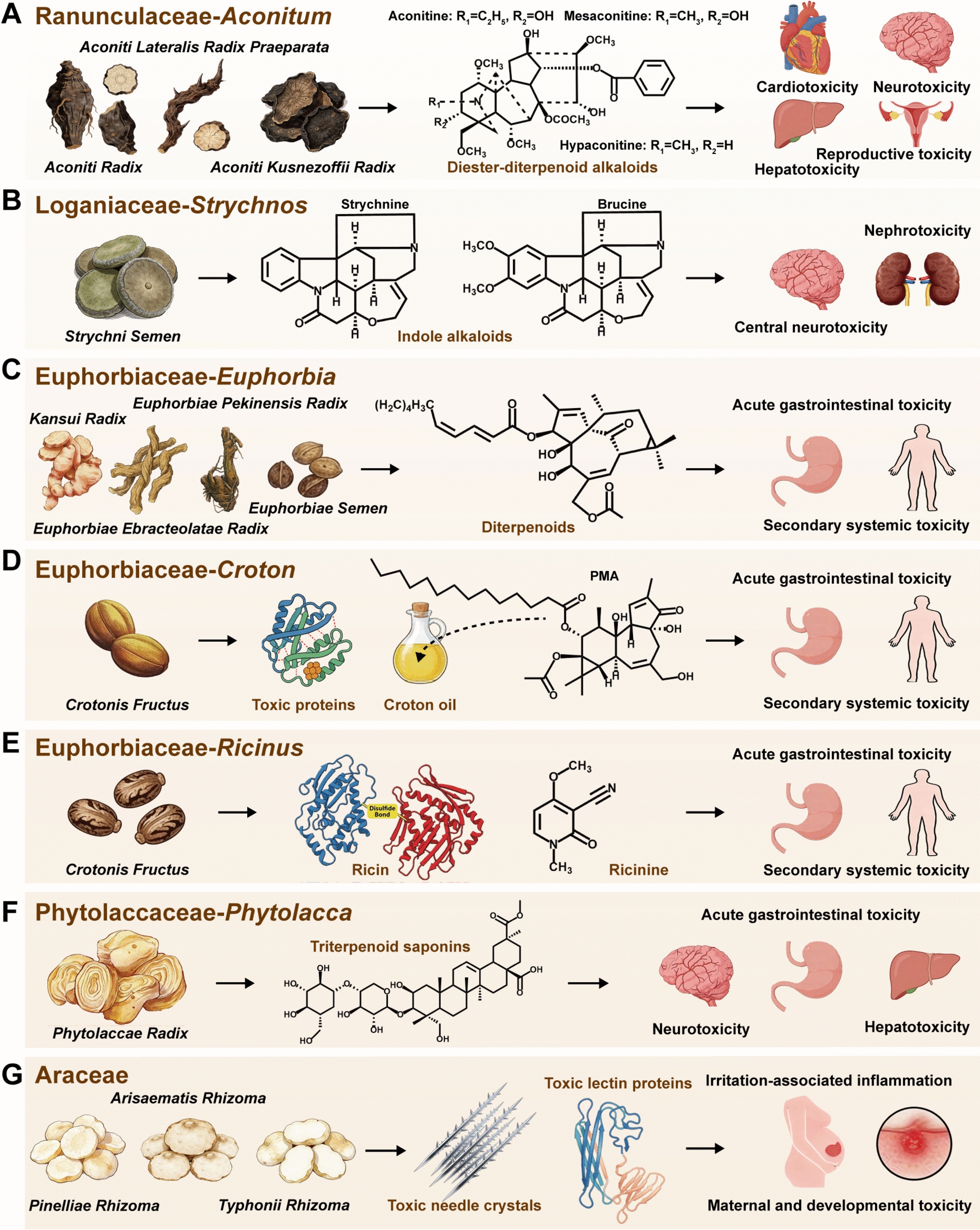
Characteristic toxic constituents and associated toxic manifestations of representative plant-derived toxic TCMs. **A** Family Ranunculaceae. **B** Family Loganiaceae. **C** Genus *Euphorbia*, Family Euphorbiaceae. **D** Genus *Croton*, Family Euphorbiaceae. **E** Genus *Ricinus*, Family Euphorbiaceae. **F** Family Phytolaccaceae. **G** Family Araceae

The Ranunculaceae family constitutes a critical group in the study of toxic TCMs. Notably, *Aconitum* species such as *Aconiti Radix Lateralis Praeparata* (Fuzi), *Aconiti Radix* (Chuanwu), and *Aconiti Kusnezoffii Radix* (Caowu), are renowned for their therapeutic effects against conditions like rheumatoid arthritis. However, their pronounced toxicity means that improper processing, incorrect use, or excessive dosage can readily lead to acute poisoning and even fatality [21, 22]. This toxicity is primarily attributed to their diester-diterpenoid alkaloids (DDAs), including aconitine, hypaconitine, and mesaconitine [23]. The core acute toxic manifestations of these constituents are cardiotoxicity and neurotoxicity [24], with the sustained activation of voltage-gated sodium (Na^+^) channels serving as their common molecular initiating event. This activation triggers a cascade of pathological processes, including intracellular ion homeostasis collapse, energy metabolism dysregulation, oxidative stress, and inflammatory responses, ultimately resulting in apoptosis and electrophysiological dysfunction of cardiomyocytes and neurons. Clinically, these manifestations often present as acute poisoning symptoms such as arrhythmias, neural paralysis, and respiratory-circulatory failure [25]. Specifically, in cardiomyocytes, persistent Na^+^ influx induces Na^+^/Ca^2+^ overload, which directly drives electrophysiological disturbances and fatal arrhythmias. Concurrently, it triggers mitochondrial oxidative stress, suppresses the key regulator PGC‑1α, and impairs ATP synthesis, leading to energy metabolism collapse. Moreover, this cascade activates the NLRP3 inflammasome, precipitating inflammatory apoptosis in cardiac cells [26, 27]. In neurons, sustained activation of Na^+^ channels induces continuous membrane depolarization, not only disrupting electrical signal transmission but also, via excitotoxic pathways, promoting excessive release of excitatory amino acids such as glutamate. This exacerbates Ca^2+^ influx, resulting in intracellular Ca^2+^ overload, hyperactive glycolysis, lactate accumulation, oxidative stress, and mitochondrial damage, ultimately culminating in neuronal apoptosis mediated by mitochondrial apoptotic pathways involving Bax/Bcl‑2 and related factors [28–30]. In addition to their acute toxicity, modern toxicological studies indicate that these alkaloids may exert other toxic effects. In the liver, they can induce hepatotoxicity via mechanisms including disruption of metabolic homeostasis, induction of oxidative stress and inflammatory responses, and interference with bile acid homeostasis [31–33]. In the reproductive system, they may demonstrate embryotoxicity both by interfering with gene programs essential for embryonic development and by inducing DNA damage [34] (Fig. 1A). To reduce the toxicity of these TCMs, traditional processing methods primarily involve decocting or steaming with water, or co-processing with adjuvants (e.g., Danba solution, licorice, and black soybean), aiming to decrease the content of highly toxic DDAs, thereby achieving the purpose of toxicity reduction while preserving efficacy [35].

#### Family Loganiaceae

*Strychni Semen* (Maqianzi), a representative toxic TCM of the Loganiaceae family, exhibits pharmacological activities such as anti‑inflammatory, analgesic, and antitumor effects [36]. In clinical practice, it is commonly used to treat rheumatoid arthritis, muscular pain, and numbness [37]. However, due to its potent toxicity, its clinical application requires extreme caution. Both its pharmacological activity and toxicity are primarily attributed to its indole alkaloid constituents, among which strychnine and brucine are the most critical [38, 39]. Strychnine accounts for approximately 40–50% of the total alkaloids in *Strychnos Semen* (Maqianzi) and possesses exceptionally high toxicity. Brucine, which is present at a slightly lower concentration and exhibits relatively lower toxicity, demonstrates greater pharmacological efficacy in analgesia and anti‑inflammation [40]. These alkaloids are lipid-soluble, enabling rapid systemic distribution and efficient penetration across the blood–brain barrier, thereby conferring highly selective toxicity against the central nervous system—a major limitation for their clinical use. Studies indicate that the core mechanism underlying the neurotoxicity of strychnine and brucine involves their potent competitive antagonism of glycine receptors, thereby specifically blocking glycine-mediated postsynaptic inhibition in the spinal cord and brainstem [41]. This leads to disinhibition and hyperexcitability of motor neurons, resulting in generalized tonic–clonic convulsions. Furthermore, studies suggest that these alkaloids may also directly induce programmed cell death in neurons and glial cells via activation of the PPARγ/NF-κB/caspase-3-dependent apoptotic pathway, offering a novel explanation for their potential long-term or subacute neurotoxic effects [42]. In addition to central neurotoxicity, these alkaloids can accumulate in renal cells and disrupt tyrosine metabolism, leading to nephrotoxicity that may manifest clinically as renal impairment, acute renal failure, and secondary rhabdomyolysis [43, 44]. Notably, the toxicity of *Strychnos Semen* (Maqianzi) is strongly influenced by both dosage and route of administration [45]. The unprocessed herb exhibits severe toxicity and is generally restricted to topical application; however, due to the transdermal absorption of its lipophilic toxic constituents, large-area external application should be avoided. For oral administration, the herb must be processed by sand roasting until it swells and turns brown or dark brown, or be combined with other herbs such as *Glycyrrhizae Radix* (licorice) to reduce toxicity, while its dosage must be strictly controlled within a safe therapeutic range [46] (Fig. 1B).

#### Family Euphorbiaceae

The Euphorbiaceae family constitutes an important group of toxic TCMs, represented by species such as *Kansui Radix* (Gansui), *Euphorbiae Pekinensis Radix* (Jingdaji), *Euphorbiae Ebracteolatae Radix* (Langdu), and *Euphorbiae Semen* (Qianjinzi) from the genus *Euphorbia*, as well as *Crotonis Fructus* (Badou) from the genus *Croton*, and *Ricini Semen* (Bimazi) from the genus *Ricinus*. These TCMs share common features: potent toxicity, a narrow therapeutic window, and strong therapeutic actions that readily impair spleen and stomach function. Their acute toxicity begins with direct damage to the gastrointestinal mucosa caused by highly irritant constituents [47]. Upon oral ingestion, these constituents rapidly trigger severe inflammatory responses in the gastrointestinal tract, clinically manifested as intense vomiting, abdominal pain, and either watery or hemorrhagic diarrhea. Without prompt intervention, substantial fluid loss can lead to systemic intoxication symptoms such as dehydration, electrolyte imbalance, and circulatory collapse, often accompanied by secondary damage to parenchymal organs including the liver and kidneys.

The toxicity of *Euphorbia* species is primarily attributed to diterpenoid constituents [48–50]. These constituents can induce extensive gastrointestinal injury and multi‑organ toxicity through diverse mechanisms, including direct cytotoxicity, induction of oxidative stress, activation of inflammatory responses, and disruption of key cellular signaling pathways. For example, the diterpenoid constituent 3-*O*-(2’*E*,4’*Z*-decadienoyl)-20-*O*-acetylingenol in *Kansui Radix* (Gansui) can induce oxidative stress by increasing malondialdehyde (MDA) levels, reducing glutathione (GSH) content, suppressing superoxide dismutase (SOD) activity, and impairing mitochondrial function. Concurrently, it upregulates pro-inflammatory cytokines such as IL-2 and IL-8, and activates caspase-3 and caspase-9, thereby triggering inflammatory responses and promoting apoptosis [51]. The intestinal toxicity of diterpenoid constituents in *Euphorbiae Pekinensis Radix* (Jingdaji) has been well‑documented, with major mechanisms involving disruption of aquaporin expression, induction of apoptosis, and perturbation of metabolic homeostasis. The representative constituent pekinenin C activates the NF‑κB signaling pathway, upregulating the expression of aquaporin-3 (AQP3) and its corresponding mRNA in HT‑29 cells, thereby disturbing intestinal water‑electrolyte homeostasis and leading to diarrhea [52, 53]. Moreover, pekinenin C not only induces apoptosis in intestinal epithelial cells via mitochondrial and death receptor‑dependent pathways, but also arrests the cell cycle and suppresses DNA synthesis, promoting apoptosis prior to cell division [54]. Similarly, another constituent, pekinenal, induces intestinal toxicity through analogous mechanisms while also disrupting fatty acid oxidation and phospholipid/sphingolipid metabolism, thereby exacerbating hepatocyte apoptosis and liver injury [55, 56] (Fig. 1C).

Notably, as a seed‑based TCM, *Euphorbiae Semen* (Qianjinzi) contains abundant fatty oil that lubricates the intestines and exacerbates diarrhea. Simultaneously, the oil acts as a lipophilic solvent that enhances the dissolution, dispersion, and absorption of diterpenoid ester toxins such as euphorbiasteroid in the gastrointestinal tract, thereby amplifying their local irritancy and systemic toxicity [57, 58]. In contrast, the toxicity of *Crotonis Fructus* (Badou) primarily stems from its high content of croton oil (approximately 40–60%), in which phorbol 12-myristate 13-acetate (PMA) acts as a potent inflammatory inducer and tumor promoter through aberrant activation of protein kinase C (PKC) signaling, triggering intense inflammatory responses [59, 60]. Furthermore, toxic proteins present in *Crotonis Fructus* (Badou), such as croton protein I and II, not only exert direct cytotoxicity by inhibiting protein synthesis but also induce hemolysis by damaging erythrocyte membranes [61, 62]. These effects synergize with the irritant constituents of croton oil, significantly aggravating systemic toxic reactions (Fig. 1D).

*Ricini Semen* (Bimazi) is also a representative toxic TCM within the Euphorbiaceae family. Its primary toxic constituent is ricin, a type II ribosome-inactivating protein (RIP) composed of A and B chains linked by a disulfide bond. The A chain exhibits N-glycosidase activity, which inactivates ribosomes, thereby inhibiting protein synthesis and exerting its toxic effects. The B chain recognizes and binds to galactose ligands on the cell surface, facilitating ricin endocytosis and intracellular internalization [63]. Ricin exerts potent direct damage to the gastrointestinal mucosa. Upon oral administration, it induces severe hemorrhagic enteritis, multiple organ failure, and even death [64]. Its toxicity is extremely potent, far exceeding that of other toxic constituents within the same family, and no effective treatment is currently available for ricin poisoning. Besides ricin, *Ricini Semen* (Bimazi) also contains small amounts of other toxic constituents such as ricinine [65] (Fig. 1E).

In summary, toxic TCMs from the Euphorbiaceae family share a common toxicological phenotype of severe gastrointestinal irritation, with diterpenoids, toxic oils, and toxic proteins serving as the major toxic substances. Consistent with the traditional processing theory that “unprocessed TCMs are potent and toxic, whereas processed ones are milder and less toxic”, these TCMs must be processed using methods such as vinegar frying, defatting to produce frost, or heat treatment prior to clinical use, to reduce acute toxicity and ensure medication safety.

#### Family Phytolaccaceae

*Phytolaccae Radix* (Shanglu), a representative toxic TCM derived from the Phytolaccaceae family, acts as a potent hydragogue cathartic. It is clinically employed primarily for excess syndrome characterized by severe edema and obstruction of urination and defecation—for example, ascites in liver cirrhosis, edema in nephrotic syndrome, and pleural effusions—to rapidly eliminate water and reduce swelling [66, 67]. However, its therapeutic window is extremely narrow, with the effective dose lying close to the toxic threshold. Inappropriate administration can readily induce acute gastrointestinal injury, manifested as vomiting, abdominal pain, and diarrhea, and may trigger systemic inflammatory responses that progress to multi-organ dysfunction [68]. Therefore, it is strictly contraindicated in patients with constitutional deficiency, spleen-stomach weakness, or renal insufficiency. The potent gastrointestinal irritation and systemic toxicity of *Phytolaccae Radix* (Shanglu) are closely associated with its triterpenoid saponin constituents [69]. Studies have confirmed that phytolaccoside A, B, C, and H exert local irritant effects on the mucosa and can induce macrophages to release key inflammatory mediators including NO, TNF‑α, and IL‑1β, thereby triggering intense inflammatory cascades [70]. Furthermore, phytolaccoside B exhibits neurotoxicity in larval zebrafish models, impairing central nervous system development by inhibiting acetylcholinesterase activity and disrupting cholinergic signaling [71]. Regarding hepatotoxicity, studies based on zebrafish and L‑02 cell models have further revealed that total saponins of *Phytolaccae Radix* (Shanglu) and phytolaccoside A can induce oxidative stress and disrupt energy metabolism, subsequently activating the p53 pathway to mediate apoptosis and cell‑cycle arrest, while also synergistically driving ferroptosis, ultimately leading to hepatocyte death and structural liver damage [72, 73] (Fig. 1F). Modern processing strategies for *Phytolaccae Radix* (Shanglu) should therefore aim to reduce the content of toxic triterpenoid saponins and promote their chemical transformation into less‑toxic or non‑toxic derivatives. In line with this objective, traditional processing theory requires that *Phytolaccae Radix* (Shanglu) must be used after vinegar frying, which reduces the content of toxic triterpenoid saponins, moderates its drastic purgative property, and thereby achieves toxicity reduction.

#### Family Araceae

Representative toxic TCMs of the Araceae family, including *Pinelliae Rhizoma* (Banxia), *Arisaematis Rhizoma* (Tiannanxing), and *Typhonii Rhizoma* (Baifuzi), exhibit potent irritant effects on local tissues. Upon oral ingestion, their irritant constituents rapidly act on the oral and pharyngeal mucosa, causing numbness of the mouth and tongue, throat swelling and pain, hoarseness, and excessive salivation. Severe cases may lead to asphyxia due to laryngeal edema [74]. When irritating the gastric mucosa, these substances can induce violent vomiting, abdominal pain, and other symptoms of acute gastroenteritis. The irritant toxicity is closely linked to the specialized needle-crystal complexes, known as “toxic needle crystals”. These needle crystals are primarily composed of calcium oxalate and proteins, appearing as elongated, sharply‑pointed, rigid structures with distinct barbs and grooves on the surface [75]. Modern studies have confirmed that the toxicity of Araceae TCMs primarily originates from mechanical injury caused by these toxic needle crystals and chemical damage caused by lectins [76–78]. The unique morphology of the needle crystals enables them to pierce mucosal epithelial cells, inflicting direct physical injury. Furthermore, upon penetrating tissues, toxic proteins such as lectins are rapidly released from the needle crystals and invade the intercellular space, activating macrophages and inducing oxidative stress. This subsequently promotes the massive release of inflammatory cytokines, including TNF‑α and IL‑1β, through signaling pathways such as NF‑κB, MAPK, and inflammasome activation, thereby triggering inflammatory cascades [79–81]. In addition to the acute irritant toxicity, these TCMs possess maternal and developmental toxicity, which can cause embryo resorption, growth retardation, structural malformations, and even abortion. Therefore, their use is strictly contraindicated in pregnant women and must be meticulously avoided in clinical practice [82, 83] (Fig. 1G). According to traditional processing theory, toxic Araceae TCMs must be processed with adjuvants such as ginger and alum before clinical use to moderate their drastic nature and ensure clinical safety [83].

#### Other plant-derived Toxic TCMs

Beyond the previously mentioned representative toxic families, several other plant families also contain notable toxic TCMs. Alkaloids represent one of the most common types of toxic constituents in these TCMs. Among them, tropane alkaloids (e.g., atropine, hyoscyamine, and scopolamine) exhibit distinct family-specific distribution patterns and are mainly found in Solanaceae TCMs such as *Hyoscyami Semen* (Tianxianzi), *Physochlainae Radix* (Huashanshen), and *Daturae Flos* (Yangjinhua) [84–86]. Notably, *Erycibes Caulis* (Dinggongteng) from the Convolvulaceae family also produces structurally similar tropane alkaloids [87]. As competitive antagonists of muscarinic acetylcholine receptors, these alkaloids induce a classic “atropine-like” poisoning syndrome, clinically characterized by dry mouth, flushed skin, mydriasis, tachycardia, and a range of central excitatory symptoms (e.g., restlessness, hallucinations, convulsions). Severe cases may progress to respiratory center paralysis and death. Isoquinoline alkaloids are widely distributed across multiple families, including Papaveraceae (e.g., *Chelidonii Herba*, Baiqucai; *Papaveris Pericarpium*, Yingsuke), Rutaceae (e.g., *Euodiae Fructus*, Wuzhuyu; *Zanthoxyli Radix*, Liangmianzhen), and Menispermaceae (e.g., *Menispermi Rhizoma*, Beidougen), although their skeletal types vary considerably among different families. For example, although both *Zanthoxyli Radix* (Liangmianzhen) and *Menispermi Rhizoma* (Beidougen) contain isoquinoline alkaloids, the former is dominated by benzophenanthridine-type alkaloids (e.g., nitidine chloride) [88], whereas the latter is characterized by bisbenzyltetrahydroisoquinoline-type alkaloids (e.g., dauricine and sinomenine) [89, 90]. *Euodiae Fructus* (Wuzhuyu) contains indole alkaloids (e.g., evodiamine and rutaecarpine) as well as quinolinone alkaloids (e.g., evocarpine) [91]. The alkaloids in *Papaveris Pericarpium* (Yingsuke) are mainly of the opium type (e.g., morphine and codeine), which exhibit strong addictive potential, and excessive use can readily lead to nervous system disorders [92]. In addition to alkaloids, toxic TCMs from other families and genera also contain various other types of toxic constituents. For example, *Armeniacae Semen Amarum* (Kuxingren) from the Rosaceae family mainly contains cyanogenic glycosides (e.g., amygdalin) [93, 94]; *Genkwa Flos* (Yuanhua) from the Thymelaeaceae family contains diterpenoid orthoesters (e.g., yuanhuacine) [95]; the toxicity of *Meliae Cortex* (Kulianpi) and *Toosendan Fructus* (Chuanlianzi) from the Meliaceae family is primarily attributed to toosendanin [96, 97]; and *Entadae Semen* (Ketengzi), *Gleditsiae Sinensis Fructus* (Dazaojiao), and *Gleditsiae Fructus Abnormalis* (Zhuyazao) from the Fabaceae family contain toxic saponins as their main toxic substances [98].

For most of the above‑mentioned toxic TCMs, systematic studies on processing methods and detoxification mechanisms are lacking, and relevant research remains preliminary. No recognized detoxification methods or technical systems have been established, and the *Chinese Pharmacopoeia* (2025 Edition) does not include standard detoxification procedures for these TCMs. Nevertheless, a common strategy currently employed is to utilize the thermal instability of their toxic constituents. High‑temperature treatments (e.g., stir‑frying, simmering, steaming, boiling) can directly decompose or disrupt the structure of toxic constituents, or promote their conversion into less toxic forms, thereby reducing acute toxicity and adverse reactions. This common processing strategy provides a valuable reference for subsequent investigations into the scientific basis of processing-induced detoxification, particularly from the perspective of chemical transformation.

### Animal-derived toxic TCMs

Animal-derived toxic TCMs constitute a distinctive category within the TCM system, as their toxicity largely originates from specific bioactive substances that animals have evolved for defense against predators, predation, or competition. Unlike plant‑derived toxic constituents, which are primarily secondary metabolites such as alkaloids and glycosides, the main toxic constituents of animal-derived toxic TCMs are highly bioactive proteins or peptide toxins, along with steroidal constituents such as bufogenins. For example, the principal toxic constituents of *Hirudo* (Shuizhi), *Scorpio* (Quanxie), and snake‑derived TCMs, such as *Deinagkistrodon* (Qishe) and *Bungarus Parvus* (Jinqiɑnbɑihuɑshe), are peptide/protein toxins and various bioactive enzymes [99–101]. These constituents, functioning as “high‑efficiency biological weapons” evolved through natural selection, are characterized by high potency and target specificity. The bufadienolide constituents in *Bufonis Venenum* (Chansu), such as bufalin, cinobufotalin, cinobufagin and resibufogenin [102], share a mechanism of action similar to that of cardiac glycoside drugs (e.g., digoxin). By inhibiting Na^+^/K^+^-ATPase activity, they induce intracellular calcium overload, and excessive administration can lead to severe arrhythmias or even cardiac arrest, representing a high clinical risk [103, 104]. For the above‑mentioned animal-derived toxic TCMs, traditional processing methods all follow the core principle of “toxicity reduction while preserving efficacy”. The classic processing method for *Hirudo* (Shuizhi) is stir‑frying with heated talcum powder: the segments of *Hirudo* (Shuizhi) are stir‑fried together with heated talcum powder until they slightly swell and turn yellowish‑brown, which denatures and inactivates toxic proteins such as hirudin, significantly reducing their anticoagulant activity and thereby achieving detoxification [105]. The classic processing method for snake-derived TCMs is head removal followed by wine frying, which reduces toxicity by eliminating the main toxic organs and destroying the toxic structure of snake venom proteins, while also removing the unpleasant odor. For *Bufonis Venenum* (Chansu), the crude drug is processed by macerating with white liquor until it forms a thick paste, and is then dried and pulverized for use. Therefore, high‑temperature heating is a major processing method for animal-derived toxic TCMs.

**Fig. 2:**
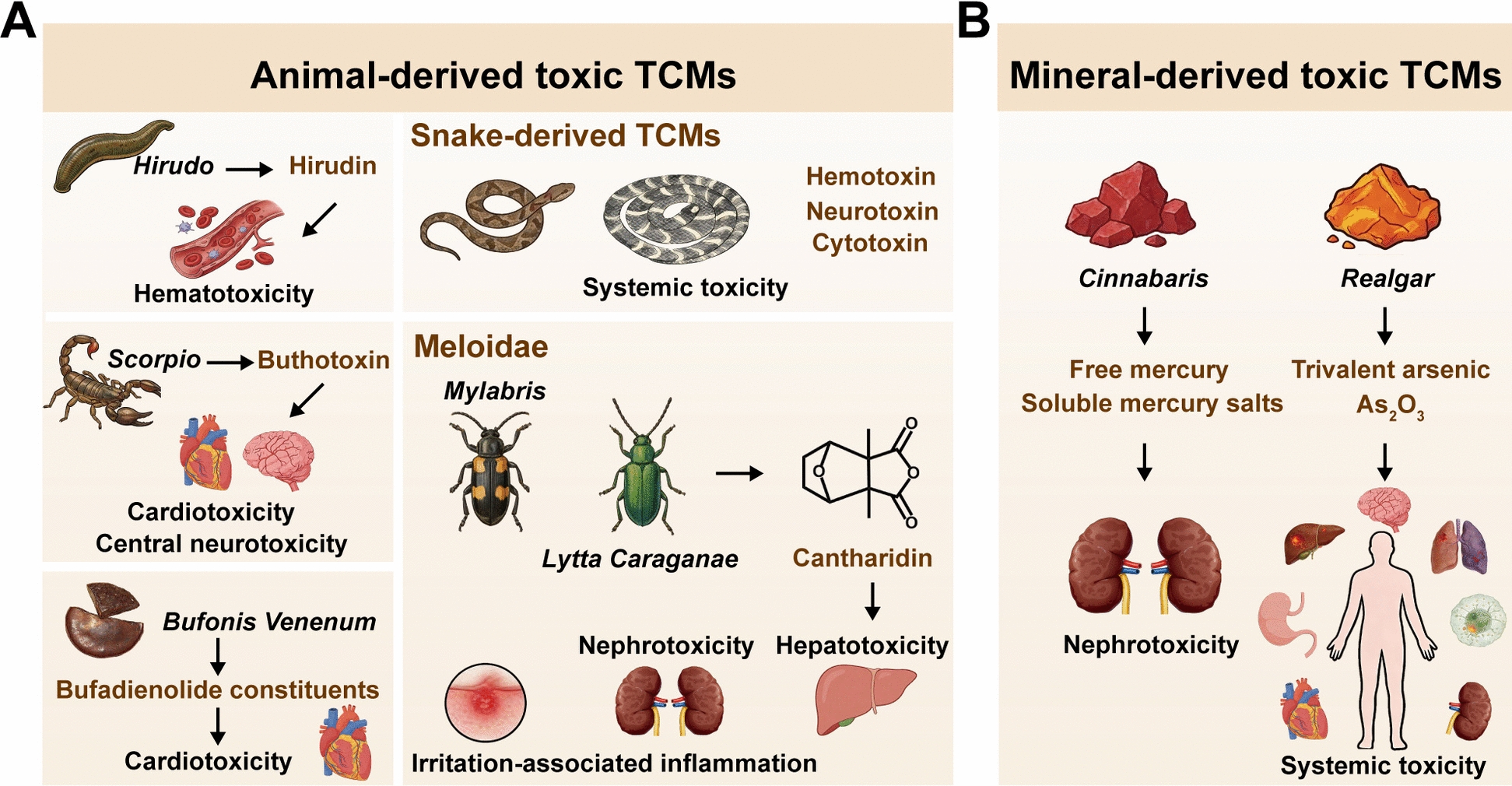
Characteristic toxic constituents and associated toxic manifestations of representative animal- and mineral-derived toxic TCMs. **A** Animal-derived toxic TCMs. **B** Mineral-derived toxic TCMs

Notably, insect-derived TCMs of the Meloidae family, including *Mylabris* (Banmao) and *Lytta Caraganae* (Qingniangzi), contain cantharidin as their main toxic constituent. Cantharidin is a highly toxic monoterpenoid that irritates the skin and mucous membranes and can cause severe damage to the kidneys and liver [106–108]. Acute renal failure and circulatory collapse are the primary causes of death from cantharidin poisoning. Studies have shown that the nephrotoxicity of cantharidin results from the synergistic action of multiple mechanisms, beginning with specific inhibition of protein phosphatase 2A and a consequent widespread disturbance of metabolic networks, involving key pathways such as tyrosine and pyrimidine metabolism. This leads to metabolite profile imbalance and hyperactive oxidative phosphorylation, thereby inducing oxidative stress. The resulting metabolic and oxidative damage further activates endoplasmic reticulum stress (especially the PERK/CHOP pathway) as well as key stress-related and metabolic signaling pathways including MAPK, AMPK, and HIF-1. These aberrant signals collectively trigger apoptosis, excessive autophagy, and inflammatory responses in renal tubular epithelial cells, ultimately leading to structural renal damage and functional impairment [109–111]. In recent years, the hepatotoxicity of cantharidin has also been systematically investigated. Studies indicate that its hepatotoxicity is closely associated with metabolic dysregulation, endoplasmic reticulum stress, and mitochondrial dysfunction. Cantharidin inhibits GSH synthesis, disrupts bile acid homeostasis, and interferes with glycerophospholipid and sphingolipid metabolism, subsequently inducing endoplasmic reticulum stress and activating the TNF-α/NF-κB inflammatory pathway [112–115]. Simultaneously, it upregulates the DDIT4/mTOR signaling pathway to promote autophagy and apoptosis [116], accompanied by dysregulation of the Bcl-2/Bax balance, which triggers the mitochondrial apoptotic pathway [117]. The combined effects of metabolic disturbance, inflammatory storm, and stress signaling ultimately lead to hepatocyte apoptosis, autophagy, and necrosis, resulting in irreversible liver damage (Fig. 2A). To mitigate the potent toxicity of *Mylabris* (Banmao), the classic detoxification processing method is stir‑frying with rice: the insects are stir‑fried together with rice until the rice turns yellowish‑brown. Additionally, the head, feet, and wings are also removed to further reduce toxicity.

### Mineral-derived toxic TCMs

Mineral-derived toxic TCMs, such as *Cinnabaris* (Zhusha), *Realgar* (Xionghuang), *Sulfur* (Liuhuang), *Calomelas* (Qingfen) and *Hydrargyri Oxydum Rubrum* (Hongfen), contain elemental constituents that can cause significant harm to the human body. Compared to small-molecule toxins derived from plants, these mineral constituents often act through more direct pathways and exert stronger toxic effects. Taking mercury-containing *Cinnabaris* (Zhusha) as an example, its main constituent is mercuric sulfide (HgS), traditionally used to alleviate symptoms such as palpitations, restlessness, and insomnia [118]. However, impurities including free mercury and soluble mercury salts confer a clear toxic risk [119]. After oral ingestion, *Cinnabaris* (Zhusha) slowly releases soluble mercury ions (Hg^2+^) in the intestine. Following absorption, Hg^2+^ is transported via the bloodstream to the kidneys, where it accumulates predominantly in proximal tubular epithelial cells, making the kidney the primary target organ of its toxicity. Specifically, Hg^2+^ displays exceptionally high affinity for sulfhydryl (-SH) groups, enabling it to bind to intracellular -SH-containing enzymes and structural proteins [120]. This interaction inhibits the activity of multiple critical enzymes, including antioxidant enzymes such as GSH peroxidase and catalase, as well as mitochondrial respiratory chain-associated enzymes, leading to cellular energy metabolism dysfunction and oxidative stress. Hg^2+^ can also bind to -SH groups in cytoskeletal proteins, disrupting cellular structural integrity. Additionally, accumulated Hg^2+^ impairs the expression and function of organic anion transporters (OATs, especially OAT1 and OAT3) in the kidney, interfering with the excretion of both endogenous and exogenous toxins and thereby exacerbating renal injury [121, 122]. Furthermore, Hg^2+^ activates the NLRP3 inflammasome, triggering intense local inflammatory responses that synergistically promote the death of renal tubular epithelial cells [123]. Another representative arsenic-containing mineral medicine, *Realgar* (Xionghuang, mainly composed of As_2_S_2_), primarily exerts toxicity through trivalent arsenic constituents (e.g., As_2_O_3_). As^3+^ also possesses strong affinity for -SH groups and can induce systemic multi-organ toxicity by inhibiting -SH-dependent enzyme activity, inducing oxidative stress, and directly damaging cellular structures [124–126] (Fig. 2B). The traditional processing method for both of the above-mentioned toxic mineral-derived TCMs is water levigation, which removes soluble toxic impurities through repeated grinding and washing, thereby reducing toxicity. In addition, *Sulfur* (Liuhuang) also possesses considerable toxicity, which mainly originates from heavy metal impurities such as arsenic (As) and from sulfur dioxide (SO_2_) generated during heating. *Sulfur* (Liuhuang) intended for oral administration must be processed by boiling with tofu, whereas the crude form is restricted to external use only.

### Common principles and mechanisms underlying detoxification in processing of toxic TCMs

Processing is a critical step in reducing the toxicity of TCMs. It exerts its effects through thermal interventions (e.g., stir‑frying, steaming, boiling, and other heat treatments), adjuvant‑based interventions (e.g., liquid adjuvants such as wine and vinegar, as well as solid adjuvants including rice, licorice, and tofu), and mechanical interventions (e.g., cutting, washing). These intervention modalities often act synergistically to promote the physical removal of toxic constituents, the changes in the physical properties of decoction pieces, the chemical transformation of toxic constituents, and the regulation of their in vivo processes, thereby achieving toxicity reduction.

### Common physical detoxification mechanisms based on the separation, purification, adjuvant adsorption, and heat‑induced protein denaturation of toxic constituents

Physical detoxification methods for toxic TCMs do not rely on chemical reactions. Instead, they achieve toxicity reduction by separating and removing toxic constituents, eliminating toxic parts, or disrupting the spatial structure and physical state of toxic constituents. The core physical mechanisms can be summarized as separation and removal, purification and refinement, adjuvant adsorption, and heat-induced protein denaturation.

#### Separation and removal

**Fig. 3:**
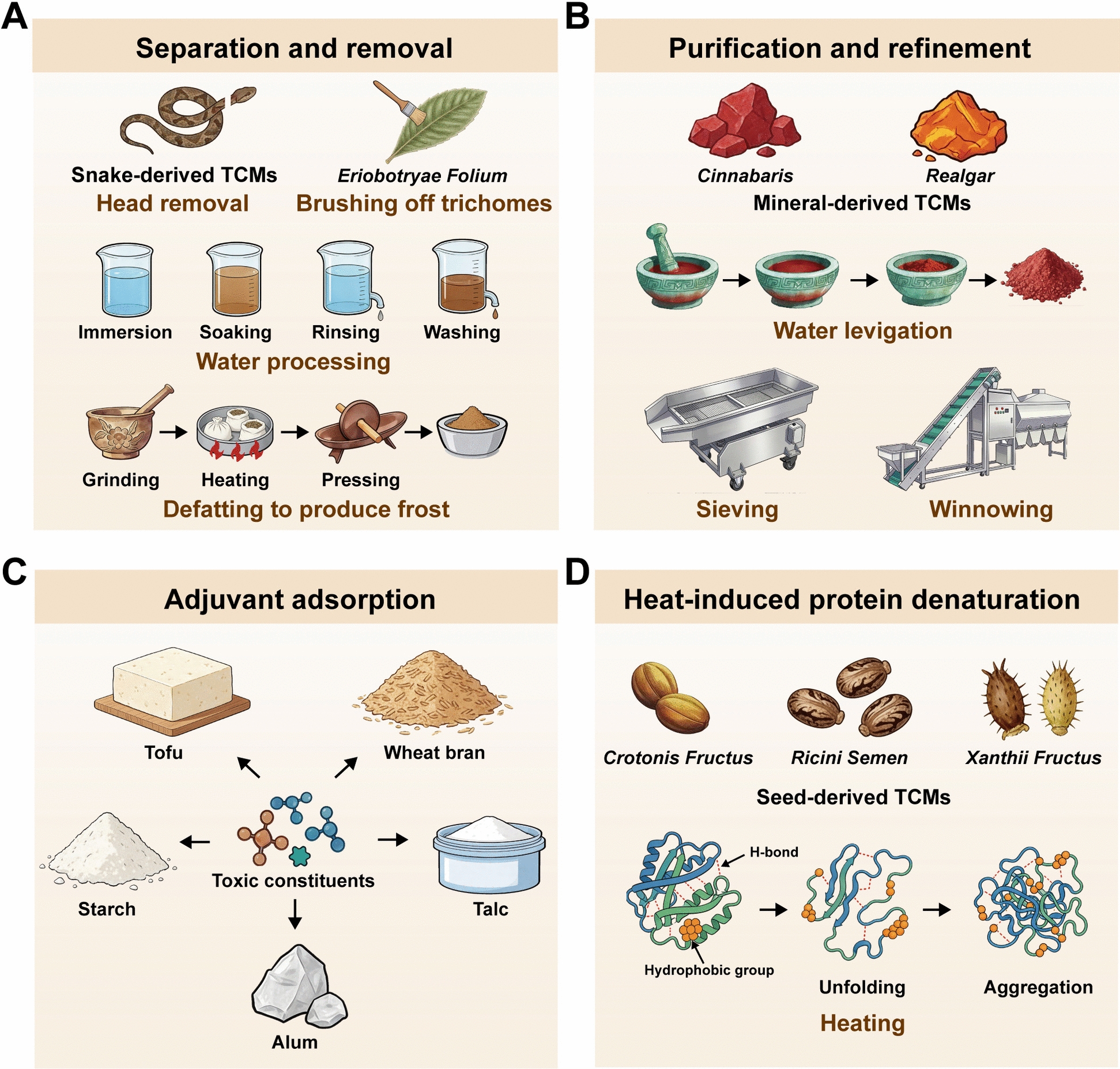
Schematic illustration of the common physical detoxification mechanisms. **A** Separation and removal. **B** Purification and refinement. **C** Adjuvant adsorption. **D** Heat-induced protein denaturation

Separation and removal aim to directly reduce the absolute content of toxic constituents in TCMs through physical separation methods. In certain TCMs, toxic constituents are predominantly concentrated in specific parts and removing these parts can effectively mitigate toxicity. For example, the heads of snake‑derived TCMs such as *Deinagkistrodon* (Qishe) and *Ptyas dhumnades* (Wushaoshe) are rich in hemorrhagic toxins and neurotoxins, which can lead to visceral bleeding and even fatality; therefore, the heads are removed before clinical use. Similarly, during the processing of *Mylabris* (Banmao), the head, legs, and wings are removed to reduce toxicity, as these parts contain higher levels of cantharidin. Likewise, the dense trichomes on the abaxial surface of *Eriobotryae Folium* (Pipaye) cause intense mechanical irritation to the throat and respiratory tract, readily inducing severe coughing and laryngeal edema; consequently, these trichomes are brushed off during processing [127]. The methods described above primarily rely on mechanical forces to separate toxic parts from the non-toxic parts. In contrast, the widely employed water-processing techniques utilize differences in the solubility or dispersibility of toxic and therapeutic constituents in water. Through procedures such as soaking, immersion, rinsing, and washing, this approach selectively promotes the transfer of toxic substances from TCMs into the water, thereby reducing toxicity. For example, prolonged rinsing of *Pinelliae Rhizoma* (Banxia), *Arisaematis Rhizoma* (Tiannanxing), and *Aconiti Radix Lateralis Praeparata* (Fuzi) can partially remove their water-soluble toxic constituents (e.g., alkaloids and irritant glycosides) [14]. Additionally, for seed-derived TCMs in which toxicity is primarily attributable to oils, the defatting to produce frost is frequently used for detoxification. This process involves removing the majority of the oil content through grinding, heating, and pressing, followed by processing the residue into a loose, powder-like product. For instance, after defatting to produce frost, *Crotonis Fructus* (Badou) and *Euphorbiae Semen* (Qianjinzi) exhibit significantly reduced oil-induced severe purgation, irritation, and other toxic reactions, resulting in milder pharmacological activity that facilitates clinical dosage control and safer application [128] (Fig. 3A).

#### Purification and refinement

Purification and refinement rely on differences in physical properties (e.g., particle size, density, and solubility) between target constituents and impurities, and employ physical methods to selectively separate non‑medicinal parts or impurities, thereby obtaining medicinal fractions with higher purity and enhanced safety. A representative technique is water levigation, which is particularly applicable to mineral‑derived TCMs [129]. The method exploits differences in sedimentation behavior of solid particles in water. Through repeated grinding, decantation, and graded settling, coarse particles and sand‑like impurities are efficiently removed, while continuous washing dissolves and eliminates soluble toxic salts. This process ultimately yields ultra‑fine medicinal powders with uniform particle size, delicate texture, and significantly reduced toxicity. After being processed via water levigation, *Cinnabaris* (Zhusha) contains abundant HgS nanoparticles with sizes below 100 nm. Compared with conventional larger crystals, these nano-sized HgS particles exhibit a markedly reduced release rate of Hg^2+^ into simulated intestinal fluid, thereby decreasing the source of bioavailable toxic mercury [130]. Similarly, *Realgar* (Xionghuang) processed by water levigation also forms nano-scale particles, in which the content of highly toxic As_2_O_3_ is significantly lowered, while other arsenic-containing constituents with antitumor activity are preserved [131]. This processing technique effectively retains the antitumor efficacy of these TCMs while substantially diminishing its toxic side effects on normal tissues. In addition, sieving and winnowing are common physical purification methods that effectively remove impurities or non‑medicinal constituents based on differences in density (sieving controls particle size, while winnowing utilizes airflow separation), thereby enhancing the overall purity and safety (Fig. 3B).

#### Adjuvant adsorption

In the processing of TCMs, adjuvants are frequently employed to exert synergistic or auxiliary effects. The physical detoxification mechanism facilitated by adjuvants is mainly based on the adsorption of toxic constituents. For example, inert adjuvants with high specific surface areas—such as tofu, wheat bran, starch, and talc—can interact with toxic alkaloids and other constituents through surface adsorption or physical encapsulation [14], thereby reducing their dissolution rate during decoction and their bioavailability in vivo. During the co‑boiling of *sulfur* (Liuhuang) with tofu, certain soluble toxic substances dissolve into the water, while others precipitate upon heating and adsorb onto the tofu. Once the tofu turns dark green, both the tofu and the liquid are discarded, thereby removing the toxic constituents. During defatting to produce frost, such adjuvants can uniformly adsorb residual oils following the pressing of seed-derived TCMs, further lowering the oil content to produce a loose, frost-like product that thereby mitigates severe purgative and other toxic reactions. Furthermore, in the processing of toxic TCMs derived from the Araceae family, the hydrolysis of alum leads to the formation of aluminum hydroxide (Al(OH)_3_) colloids. These colloids may adsorb onto and coat the sharp structures of toxic needle crystals, creating a physical barrier that prevents direct contact with mucosal tissues, thus reducing their local irritant toxicity (Fig. 3C).

#### Heat-induced protein denaturation

Toxic proteins are widely distributed in seed‑derived TCMs across multiple families, including Euphorbiaceae (e.g., *Crotonis Fructus*, Badou; *Euphorbiae Semen*, Qianjinzi; *Ricini Semen*, Bimazi) and Asteraceae (e.g., *Xanthii Fructus*, Cangerzi) [75, 132]. For these toxic constituents, traditional processing primarily employs defatting to produce frost (often combined with heating) or direct heat treatment. As recorded in ancient Chinese medical classics, there is a long-standing principle that “seeds must be stir-fried before clinical use.” The core detoxification mechanism of both approaches involves the use of thermal energy to induce denaturation of toxic proteins [133, 134]. Under sustained heating, the tertiary and quaternary structures of toxic proteins undergo irreversible disruption, characterized by hydrogen bond breakage, exposure of hydrophobic groups, peptide chain unfolding, and aggregation, ultimately leading to the loss of their original toxicity and biological activity. This process directly destroys the spatial conformation and function of the toxic proteins via physical means, effectively suppressing toxic effects at their source and embodying the key scientific principle of “using heat to counteract toxicity” in traditional processing. For example, heating can inactivate the proteins in *Crotonis Fructus* (Badou), thereby significantly alleviating the resulting gastrointestinal damage (Fig. 3D).

### Common chemical detoxification mechanisms based on degradation and structural transformation of chemical constituents

Chemical detoxification of toxic TCMs refers to a systematic strategy for reducing toxicity through chemical reactions during processing, in which toxic constituents are transformed into less‑toxic or non‑toxic substances or are structurally modified to diminish their harmful effects. In contrast to physical detoxification, the core of chemical detoxification lies in the fundamental modification of the chemical structure of the toxic constituents.

#### Heat- and adjuvant-induced chemical degradation and structural transformation of toxic constituents

**Fig. 4:**
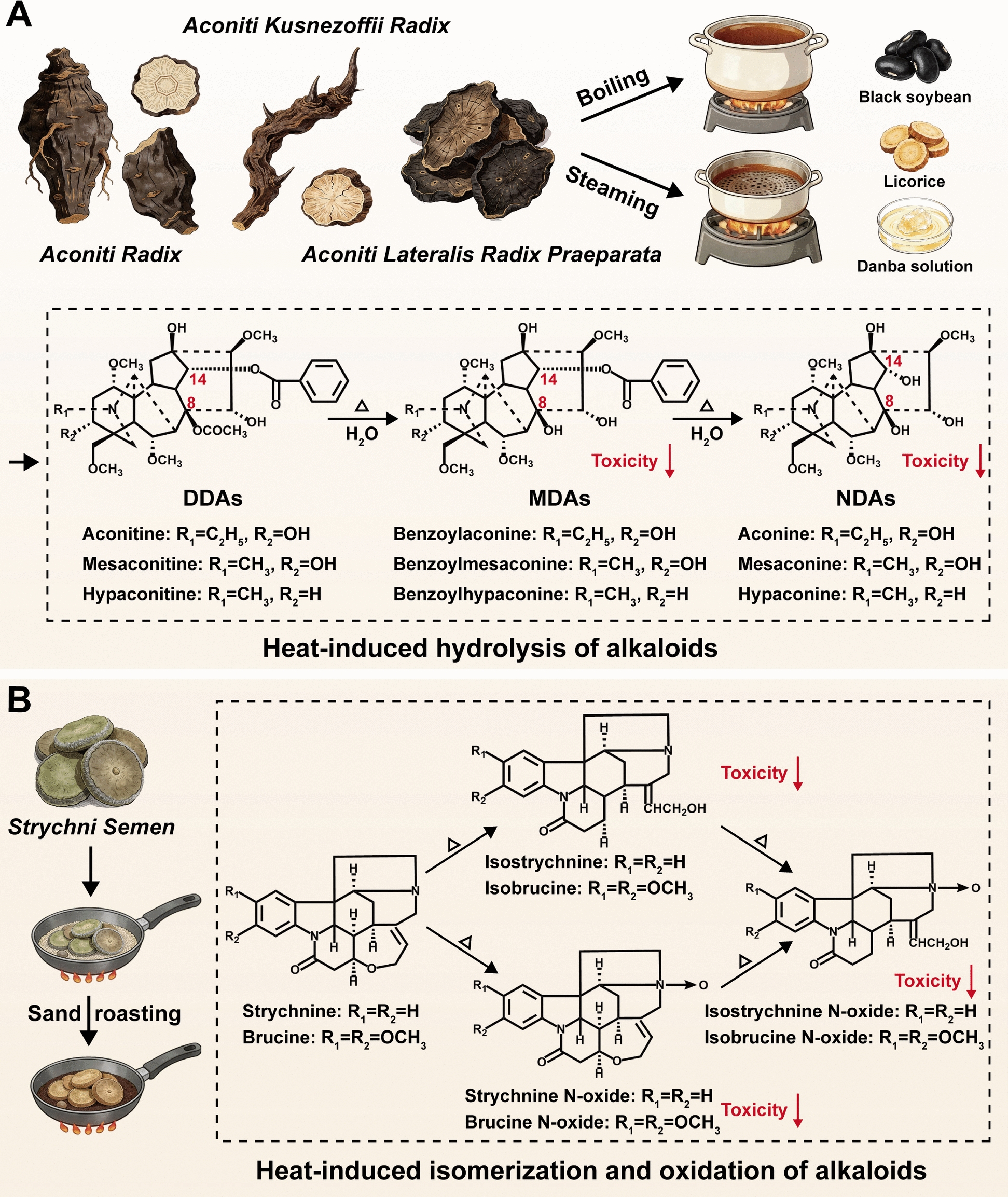
Heat‑mediated structural transformation mechanisms of alkaloids from Ranunculaceae and Loganiaceae families. **A** Heat-induced hydrolysis of diester-diterpenoid alkaloids from Ranunculaceae. **B** Heat-induced isomerization and oxidation of alkaloids from Loganiaceae

*Heat-induced hydrolysis and transesterification of alkaloids from Ranunculaceae.* TCMs containing toxic alkaloids often undergo high‑temperature processing, promoting specific chemical transformations in their alkaloid constituents. Taking Ranunculaceae TCMs such as *Aconiti Radix* (Chuanwu), *Aconiti Kusnezoffii Radix* (Caowu), and *Aconiti Radix Lateralis Praeparata* (Fuzi) as examples, their major toxic constituents are DDAs. When subjected to high‑temperature treatments such as steaming or boiling, these constituents undergo stepwise hydrolysis of ester bonds, converting them into nonester‑diterpenoid alkaloids (NDAs), which significantly reduces their toxicity [135, 136]. The specific transformation pathway is as follows: highly toxic DDAs (e.g., aconitine, mesaconitine, and hypaconitine) first undergo hydrolysis of the C_8_‑acetyl ester bond under heating, losing one molecule of acetic acid to yield the corresponding monoester‑diterpenoid alkaloids (MDAs, e.g., benzoylaconine, benzoylmesaconine, and benzoylhypaconine). These intermediates can be further hydrolyzed, with the C_14_‑benzoyl ester bond being cleaved and one molecule of benzoic acid released, resulting in the formation of NDAs (e.g., aconine, mesaconine, and hypaconine). The final products exhibit drastically reduced toxicity—only 1/2000 to 1/4000 of that of the original DDAs [137, 138] (Fig. 4A). Additionally, during heating, DDAs may also undergo transesterification, whereby the C_8_‑acetyl group is replaced by an aliphatic acyl group, producing less‑toxic lipo‑type alkaloids [139, 140]. Notably, in addition to conventional processing methods, such TCMs are also often processed with special adjuvants. For example, honey‑boiled *Aconiti Radix* (Chuanwu) has been documented in the Han‑dynasty medical classic *Synopsis of the Golden Chamber* (JinGui YaoLue) by Zhang Zhongjing. Modern research indicates that while traditional processing gradually converts highly toxic DDAs into nearly non-toxic NDAs, thereby greatly reducing toxicity, it also attenuates pharmacological activities such as analgesia and anti‑inflammation. The addition of honey, however, promotes the conversion of DDAs to MDAs while inhibiting their further hydrolysis to NDAs, thus effectively lowering the DDA content while maintaining MDA levels in *Aconiti Radix* (Chuanwu). This effect is associated with the formation of approximately 250 nm supramolecular aggregates during honey‑boiling, which encapsulate MDAs and hinder their subsequent hydrolysis, thereby preserving the pharmacologically active material basis while reducing toxicity [141]. Toxicological evidence further indicates that processing significantly mitigates the cardiotoxicity of *Aconiti Radix Lateralis Praeparata* (Fuzi). Microcalorimetric evaluation comparing the toxicity of the crude drug and its processed products, revealed that the raw form exhibited the highest toxicity, whereas Danfupian showed the lowest. Furthermore, when using the minimum dose required to induce premature ventricular contractions (PVCs) in rats as an indicator, its cardiotoxicity was markedly reduced after processing [137].In recent years, advanced analytical techniques such as direct analysis in real‑time mass spectrometry (DART-MS) and desorption electrospray ionization mass spectrometry imaging (DESI-MSI), combined with dynamic metabolomics, have been applied for real‑time monitoring of processing and spatial distribution analysis of constituents. These techniques help precisely elucidate the dynamic transformation patterns of toxic constituents and determine the optimal processing duration for converting DDAs to MDAs and NDAs [139, 142–144].

*Heat-induced isomerization and oxidation of alkaloids from Loganiaceae.* Beyond hydrolysis, toxic alkaloids can also undergo detoxification via transformation pathways such as isomerization. Isomerization refers to the structural rearrangement of a constituent without changing its molecular composition or weight. The principal toxic constituents of Loganiaceae TCM *Strychni Semen* (Maqianzi)—strychnine and brucine—undergo structural rearrangement at the C_10_ carbonyl and adjacent carbon atoms during high-temperature sand roasting, converting into their respective isomers (isostrychnine and isobrucine) [40]. This subtle stereochemical change significantly reduces the affinity of these alkaloids for receptors in the central nervous system, thereby substantially decreasing toxicity. Simultaneously, high temperature induces oxidation reactions. The tertiary amine nitrogen atoms in strychnine, brucine, and their isomers are oxidized to generate the corresponding N-oxides, such as strychnine N-oxide, brucine N-oxide, isostrychnine N-oxide, and isobrucine N-oxide [145, 146]. These oxidation products are less toxic, collectively constituting the multiple chemical pathways of detoxification in *Strychni Semen* (Maqianzi) during sand-roasting processing (Fig. 4B). Toxicological evidence further indicates that after processing, the acute toxicity of *Strychni Semen* (Maqianzi) in mice is significantly reduced, while its anti-inflammatory, analgesic, and antitumor effects are retained or even enhanced [147].

**Fig. 5:**
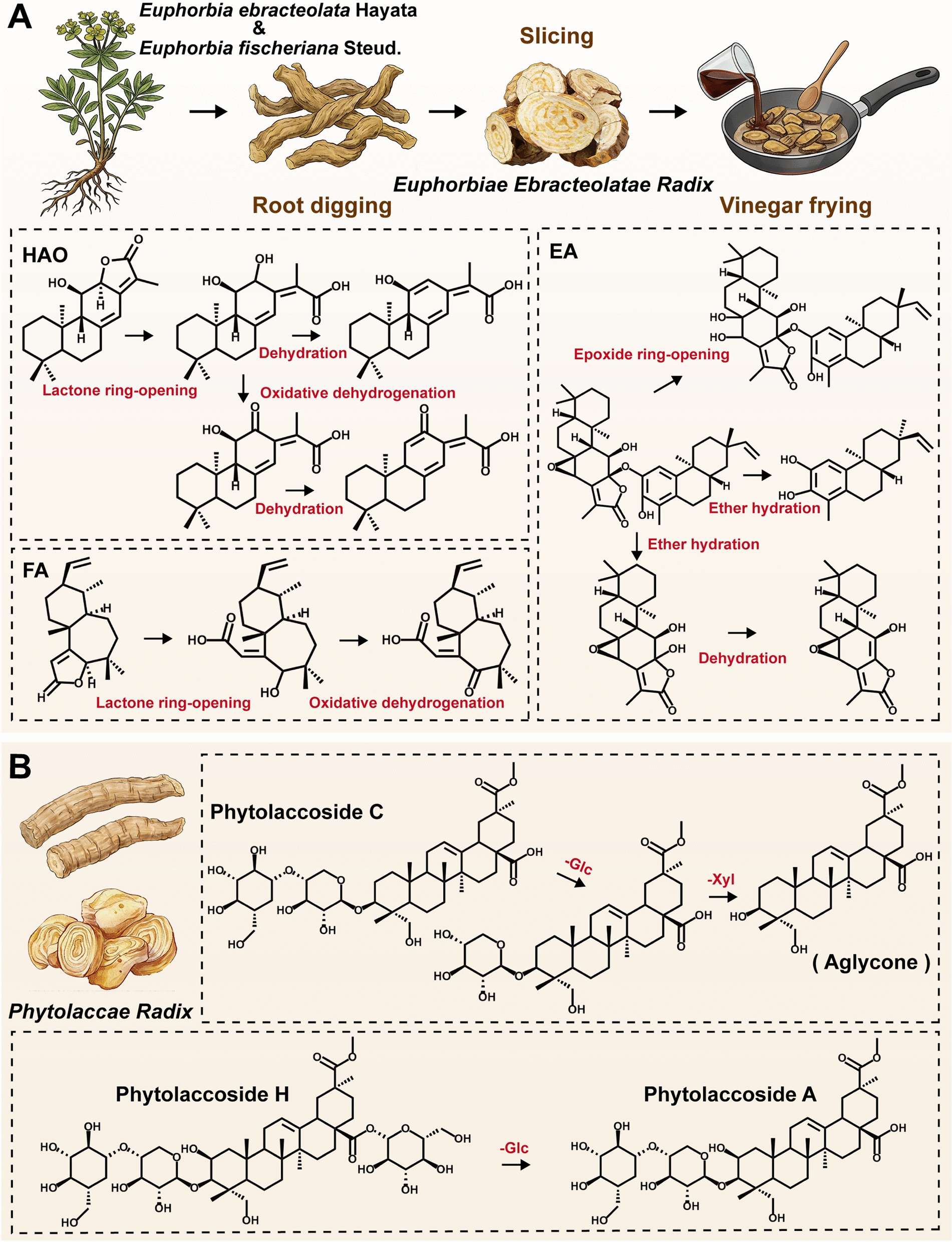
Vinegar‑frying‑mediated cleavage mechanisms of chemical bonds in Euphorbiaceae diterpenoids and Phytolaccaceae triterpenoid saponins. **A** H⁺-mediated hydrolysis and ring-opening of diterpenoids in *Euphorbiae Ebracteolatae Radix* (Langdu). **B** Vinegar-frying-mediated hydrolysis of glycosidic bonds in triterpenoid saponins of *Phytolaccae Radix* (Shanglu)

*Adjuvant H* + *-mediated hydrolysis of ester bonds and ring-opening of diterpenoid esters from Euphorbiaceae.* Terpenoids constitute a broad class of constituents widely present in TCMs. Among these, certain diterpenoids, because of their complex ring systems and specific functional groups (e.g., lactone rings, epoxide moieties, ester linkages), often exhibit marked biological activities, including cytotoxicity, cardiotoxicity, or strong gastrointestinal irritancy, thus representing an important class of toxic constituents [148–150]. The toxicity of many toxic TCMs in the Euphorbiaceae family, such as *Kansui Radix* (Gansui), *Euphorbiae Ebracteolatae Radix* (Langdu), and *Euphorbiae Pekinensis Radix* (Jingdaji), is primarily attributed to such highly irritant diterpenoid esters. Notably, vinegar frying significantly reduces the content of these toxic constituents, thereby attenuating toxicity [151, 152]. For example, vinegar-fried *Kansui Radix* (Gansui) significantly alleviates intestinal histopathological damage and inflammatory response, while also markedly improving liver function and oxidative damage markers [151]. During vinegar frying, H^+^ from vinegar serve as catalysts that protonate the carbonyl oxygen of ester bonds, promoting hydrolysis of toxic ester linkages and generating the corresponding acid and alcohol products, thereby significantly reducing toxicity. Simultaneously, under acidic conditions, unstable structural features such as lactone or epoxide rings may undergo ring-opening reactions, disrupting their original toxic conformations. Furthermore, H^+^ can facilitate dehydration of hydroxyl groups or induce skeletal rearrangements, altering molecular polarity and thus influencing solubility, membrane permeability, and bioactivity—changes that are commonly associated with diminished toxicity. For instance, abietane-type diterpene lactones in *Euphorbiae Ebracteolatae Radix* (Langdu), such as ent-11α-hydroxyabicta-8(14),13(15)-dien-16,12-olide (HAO) and Fischeria A (FA), a norditerpene lactone, can undergo lactone ring-opening during vinegar processing. When vicinal dihydroxy groups are present, further dehydration or dehydrogenation reactions may occur. Moreover, the dimeric diterpenoid Eupractenoid A (EA) can experience epoxide ring-opening and ether bond hydrolysis during vinegar frying, the latter of which predominantly cleaves the molecule into rosane-type and abietane-type diterpene monomers [153] (Fig. 5A).

*Vinegar-frying-induced hydrolysis of glycosidic bonds in triterpenoid saponins from Phytolaccaceae.* The TCMs of the Phytolaccaceae family, represented by *Phytolaccae Radix* (Shanglu), primarily contain triterpenoid saponins as their main toxic constituents. Vinegar frying can induce hydrolysis of the glycosidic bonds in these saponins, partially converting them into corresponding aglycones or secondary saponins, which markedly reduces their direct irritation to the gastrointestinal mucosa and mitigates their purgative effects—a key factor for safe clinical application. Studies have shown that after vinegar frying, the total contents of phytolaccoside B and phytolaccoside C decrease significantly, leading to a substantial reduction in overall toxicity [154]. Research on structural transformations during simulated vinegar frying indicates that phytolaccoside C undergoes hydrolysis of the C_3_-glycosidic bond under acidic heating conditions, successively losing one molecule of glucose and one molecule of xylose, ultimately converting into its aglycone. Simultaneously, the more toxic phytolaccoside H can undergo hydrolysis of the C_28_-ester glycosidic bond and release one glucose molecule, yielding the relatively less toxic phytolaccoside A, thus achieving the conversion of highly toxic constituents into less toxic ones. In summary, the chemical transformations occurring during the processing of *Phytolaccae Radix* (Shanglu) result in structural modification and targeted degradation of toxic constituents, significantly lowering the overall toxicity of the processed product [155] (Fig. 5B).

*Disruption of toxic needle crystals structure in Araceae through adjuvant‑mediated co‑processing.* Toxic TCMs belonging to the Araceae family contain calcium oxalate-lectin protein complexes termed “toxic needle crystals” as their principal toxic constituents. Consequently, the core objective of processing is to disrupt the structure of these complexes [156]. The processed forms of these TCMs include *Pinelliae Rhizoma Praeparatum Cum Alumine* (Qingbanxia), *Pinelliae Rhizoma Praeparatum Cum Zingibere Et Alumine* (Jiangbanxia), *Pinelliae Rhizoma Praeparatum* (Fabanxia), *Arisaematis Rhizoma Preparatum* (Zhitiannanxing), and *Typhonii Rhizoma Praeparatum* (Zhibaifuzi). Notably, the processing of these TCMs involves boiling with alum. Alum (KAl(SO_4_)_2_·12H_2_O) serves as a key adjuvant in the processing of Araceae TCMs; in solution, its aluminum ions (Al^3+^) can bind with oxalate ions (C_2_O_4_^2−^) from the toxic needle crystals to form aluminum oxalate complexes, leading to dissolution of calcium oxalate and causing morphological alterations such as fracturing, fragmenting, blunting, and corroding of the needle crystals, thereby abolishing their mechanical irritation to mucous membranes [83, 157]. The specific reactions underlying this process are outlined in steps (1)–(3) below. Simultaneously, soaking in alum solution induces hydrolysis of lectin proteins, alters their peptide sequences, and thereby reduces their pro-inflammatory activity [158]. Ginger juice is another common adjuvant used in the processing of Araceae TCMs. The lipophilic nature of toxic needle crystals makes them susceptible to specific denaturation by ginger extracts [159, 160]. The processing of *Pinelliae Rhizoma Praeparatum* (Fabanxia) employs licorice and limewater to establish and maintain an alkaline environment (pH > 12). This alkaline condition disrupts the unique crystalline structure of toxic needle crystals and induces hydrolysis of lectin proteins, breaking them down into small peptide fragments, thereby reducing the content of lectin proteins and achieving the goal of detoxification through processing [161, 162].

1 $$KAl\left( {SO_{4} } \right)_{2} \cdot 12H_{2} O \to K^{ + } + Al^{{3 + }} + 2SO_{4}^{{2 - }} + 12H_{2} O$$

2 $$Ca{C}_{2}{O}_{4}\left(s\right)\leftrightarrow {Ca}^{2+}+{C}_{2}{O}_{4}^{2-}$$

3 $${Al}^{3+}+3{C}_{2}{O}_{4}^{2-}\to {\left[Al{\left({C}_{2}{O}_{4}\right)}_{3}\right]}^{3-}$$

**Fig. 6:**
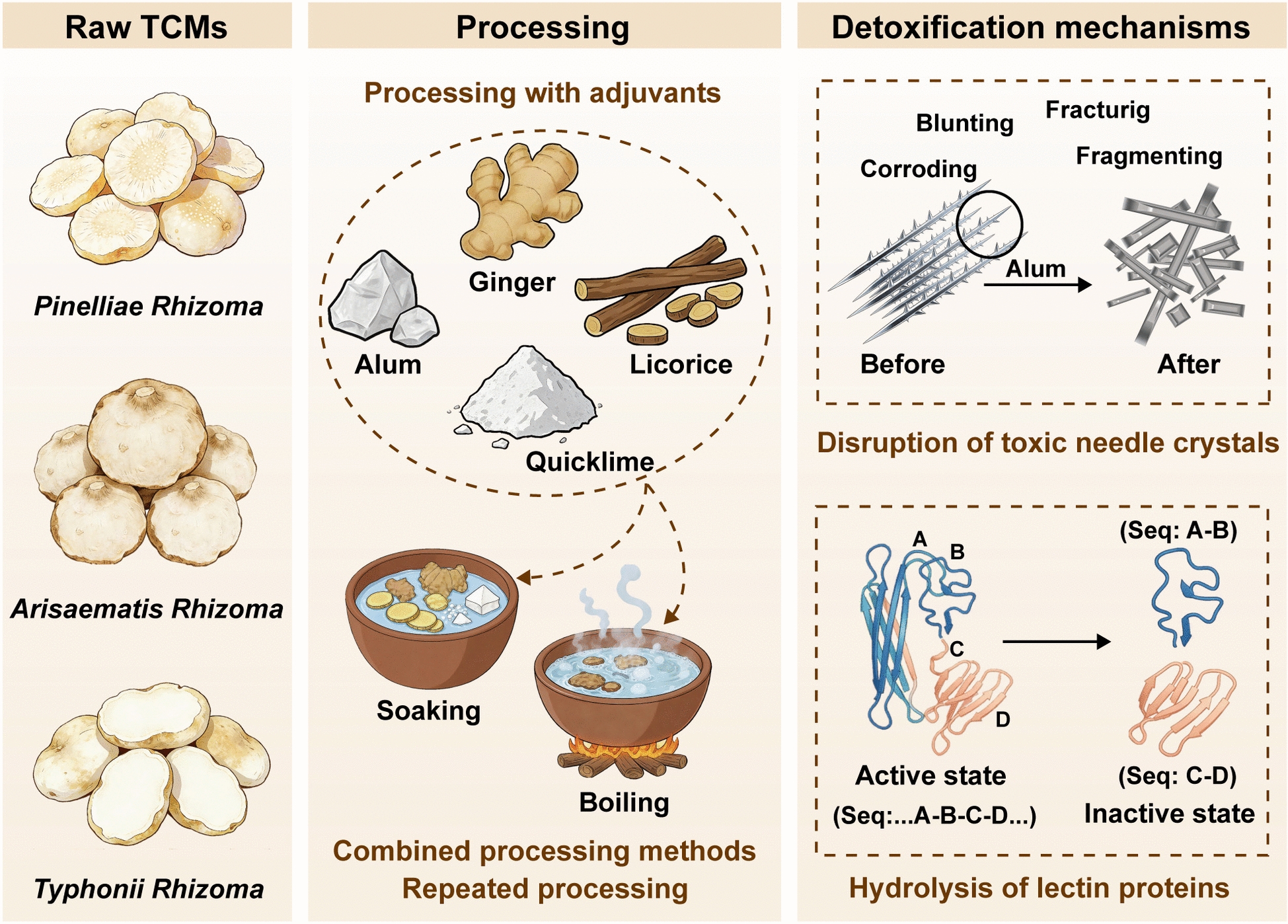
Mechanism of synergistic disruption of toxic needle crystals and lectin proteins in Araceae TCMs by co‑processing with adjuvants

Toxic needle crystals exhibit thermal stability, and conventional processing methods rely on prolonged soaking or boiling, which are cumbersome, time-consuming, prone to causing the loss of water-soluble active constituents (e.g., polysaccharides, alkaloids), and increasing the risk of material deterioration. Furthermore, Al^3+^ may pose potential health hazards, such as Alzheimer’s disease and dialysis-related encephalopathy. In response to these issues, modern research has introduced microwave irradiation, which can effectively reduce the content and alter the morphology of the needle crystals within 10 min, while better preserving active constituents. During this process, significant changes also occur in the secondary structure of lectin proteins, characterized by an increase in β‑sheet content, a decrease in β‑turn and random‑coil fractions, and the formation of insoluble aggregates, thereby offering a new approach for processing Araceae TCMs [163]. In summary, the shared detoxification mechanism for toxic Araceae TCMs involves the reaction of Al^3+^ from alum with C_2_O_4_^2−^ from calcium oxalate, which disrupts the structure of the toxic needle crystals, while heat, adjuvants, or alkaline conditions cause dissolution, hydrolysis, or denaturation of the lectin proteins on the needle crystal surface, thus achieving the goal of toxicity reduction with efficacy retention (Fig. 6).

*Heating- and alkaline aqueous treatment-induced sublimation and structural decomposition of cantharidin in Meloidae.* Insects-derived TCMs of the Meloidae family, including *Mylabris* (Banmao) and *Lytta Caraganae* (Qingniangzi), both contain cantharidin. Traditionally, rice stir-frying is used to detoxify these TCMs. Cantharidin is thermally labile, with a sublimation point of approximately 110℃. During rice stir-frying, the pan temperature is typically maintained around 130℃. This temperature range allows efficient sublimation and dissipation of cantharidin, thereby significantly lowering the absolute content of the toxic constituent, while avoiding charring of these insects due to excessive heat. This rapid, direct process constitutes the primary physical detoxification pathway in traditional rice stir-frying. Furthermore, the anhydride bond and lactone ring in the cantharidin molecule are susceptible to hydrolytic ring-opening in alkaline aqueous solutions, producing the corresponding cantharidate. Based on this mechanism, modern processing protocols often employ low-concentration pharmaceutical-grade sodium hydroxide solution to convert cantharidin into the more water-soluble sodium cantharidinate [164, 165]. This chemical transformation not only achieves more thorough detoxification but also helps preserve or even enhance the antitumor activity of cantharidin, demonstrating the value of chemical processing in reducing toxicity while improving efficacy.

#### Fermentation-driven structure transformation of toxic constituents

Fermentation-driven detoxification refers to a strategy in which TCMs are subjected to microbial or enzymatic fermentation under suitable conditions, during which microbial metabolic or enzymatic catalytic transforms toxic constituents into less-toxic or non-toxic products. Traditional fermentation methods involve mixing TCMs with substrates such as wheat flour or fermenting the material directly. For instance, after being processed into a fermented “Qu” preparation, *Pinelliae Rhizoma* (Banxia) exhibits markedly reduced irritancy, becomes milder and safer for use, and acquires enhanced functions such as improving digestion and fortifying the spleen‑stomach system. In modern processing practice, fermentation of certain toxic TCMs can lead to significant biodegradation or structural modification of their toxic constituents. During the fermentation of the herbal starter (“Yaomu”) used in the proprietary Chinese medicine Huafeng Dan, microbial activity promotes the removal of an acetic acid molecule from DDAs in *Aconiti Radix* (Chuanwu), generating MDAs—an example of microbe-driven specific detoxification [166]. Furthermore, bi-directional solid fermentation with medicinal fungi under low-moisture conditions can also regulate the synthesis and degradation of toxic constituents. After solid-state fermentation with fungi such as *Ganoderma* (Lingzhi), *Trametes robiniophila* (Huaier), and *Hericium erinaceus* (Houtou), the contents of the highly toxic strychnine and brucine in *Strychni Semen* (Maqianzi) decrease substantially, while the levels of their corresponding N-oxides increase [167].

#### Comparative analysis of chemical detoxification mechanisms among different families

During the heat‑ and adjuvant‑induced chemical degradation and structural transformation of toxic constituents, significant differences exist among toxic TCMs from different families/genera with respect to reaction types, chemical bond changes, and reaction driving forces. From the perspective of reaction types and chemical bond changes, the Ranunculaceae family primarily undergoes ester bond hydrolysis and transesterification of DDAs. The former involves stepwise cleavage of the C_8_‑acetyl ester bond and the C_14_‑benzoyl ester bond, while the latter refers to the replacement of the acetyl group by an aliphatic acyl group. In the Loganiaceae, as represented by *Strychni Semen* (Maqianzi), isomerization and oxidation reactions predominate. Isomerization involves intramolecular rearrangement of carbon‑carbon single bonds and carbon‑oxygen double bonds, whereas oxidation generates tertiary amine N‑oxides via the formation of nitrogen‑oxygen coordinate bonds. In the Euphorbiaceae, the primary reactions are ester bond hydrolysis and ring‑opening of the ring systems in diterpenoid esters, including lactone ring opening via ester bond cleavage and epoxide ring opening of ether rings. The Phytolaccaceae, represented by *Phytolaccae Radix* (Shanglu), mainly undergoes glycosidic bond hydrolysis of triterpenoid saponins under weakly acidic conditions. Notably, the detoxification mechanism of the Araceae does not rely solely on the disruption of chemical bonds (e.g., hydrolysis leading to peptide bond cleavage in lectin proteins), but also involves the formation of aluminum oxalate complexes, which dissolve and break the toxic needle crystals. For the Meloidae, cantharidin undergoes ring‑opening decomposition of its cyclic ether structure under alkaline and heating conditions, while heating also induces sublimation of cantharidin, achieving physical removal of the toxic constituent. Regarding reaction driving forces, the above processing procedures can be classified into two primary categories: “direct heat‑induced” and “adjuvant‑mediated”. Direct heat‑induced detoxification is mainly observed in the Ranunculaceae and Loganiaceae, in which alkaloids undergo ester bond hydrolysis or isomerization/oxidation under heating alone, without additional reagents. Adjuvant‑mediated detoxification is primarily seen in the Euphorbiaceae, Phytolaccaceae, and Araceae. The Euphorbiaceae relies on H^+^ from vinegar to catalyze the hydrolysis of diterpenoid ester bonds, further inducing ring‑opening of lactone or epoxide rings, representing a typical acid‑catalyzed reaction. The Phytolaccaceae also uses vinegar frying, but only provides a weakly acidic environment to promote glycosidic bond hydrolysis in triterpenoid saponins. The Araceae exhibits more diverse adjuvant‑mediated mechanisms, including ion complexation and peptide bond cleavage of lectin proteins. The Meloidae shows a dual driving force, combining heat‑induced sublimation of cantharidin with alkali‑mediated ring‑opening. Thus, the differences in processing mechanisms among families are not arbitrary but are jointly determined by the chemical skeletons (e.g., alkaloids, terpenoids, saponins), functional group reactivity (e.g., ester bonds, glycosidic bonds, cyclic ethers), and physical properties (e.g., crystallinity, sublimability) of the toxic constituents. Because of the structural similarity of toxic constituents within the same family, the detoxification mechanisms through chemical transformation exhibit significant family‑specific commonalities.

Fermentation‑based detoxification, as a distinctive strategy at the chemical transformation level, lacks pronounced family‑ or genus‑specific commonality in its processing mechanism. It relies on biocatalysis, wherein microbial enzyme systems perform highly selective catalysis to modify the structures of toxic constituents. Furthermore, fermentation proceeds under mild conditions, requiring neither high temperatures nor strong acids or bases, as the conditions are dictated by microbial growth and metabolism, thereby exemplifying the diversity of detoxification approaches.

### Common detoxification mechanisms based on regulation of the in vivo fate of toxic constituents

#### Inhibition of absorption and activation of toxic constituents

**Fig. 7:**
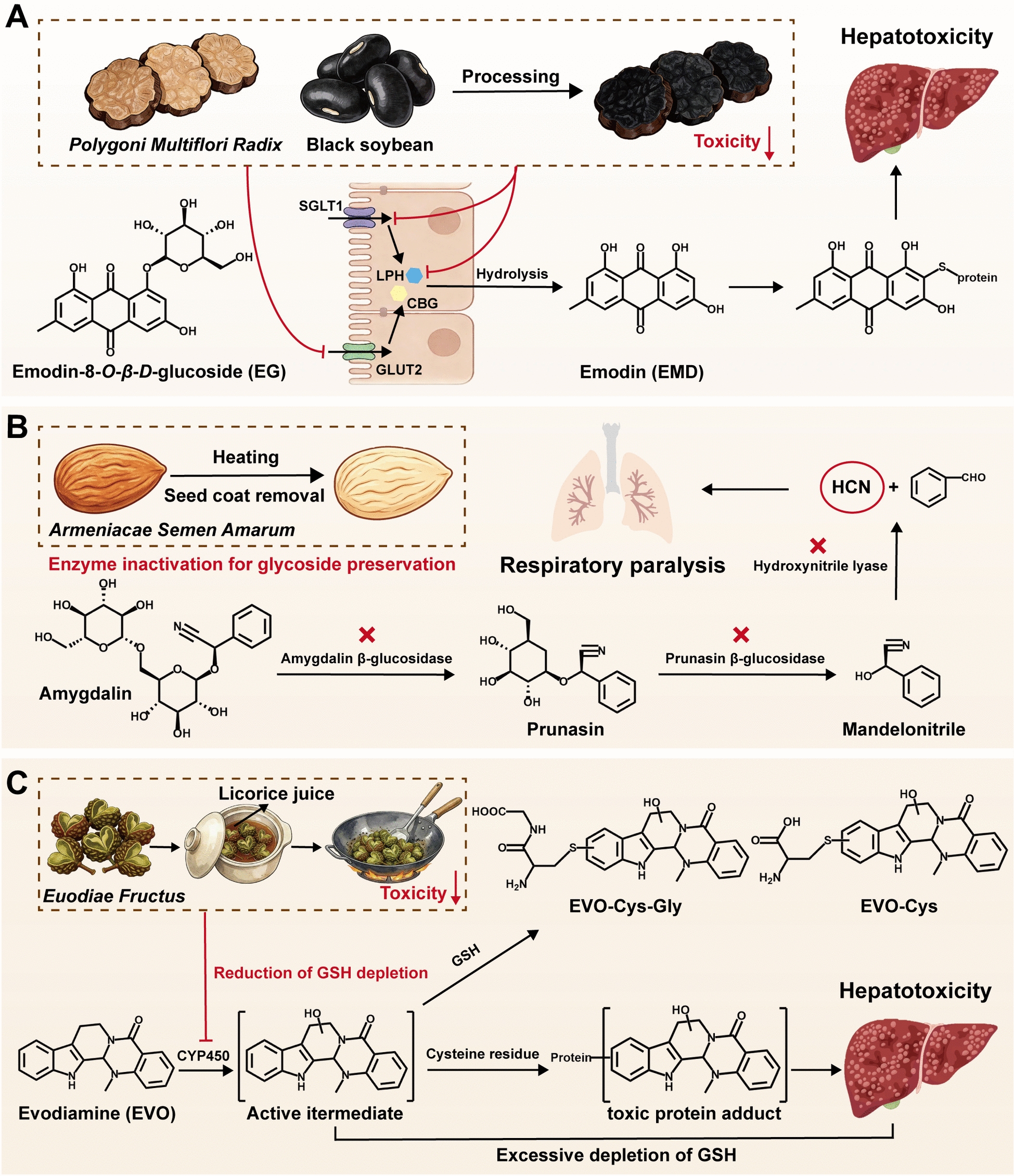
Processing-induced detoxification mechanisms based on regulation of in vivo processes: examples of *Polygoni Multiflori Radix* (Heshouwu), *Armeniacae Semen Amarum* (Kuxingren), and *Euodiae Fructus* (Wuzhuyu). **A** Mechanism of black soybean juice processing in attenuating *Polygoni Multiflori Radix* (Heshouwu) hepatotoxicity via interfering with intestinal absorption and metabolic activation of the toxic precursor. **B** Heating-induced inactivation of coexisting enzyme systems to block the hydrolysis of amygdalin to hydrogen cyanide. **C** Mechanism of licorice juice processing in attenuating *Euodiae Fructus* (Wuzhuyu) hepatotoxicity via inhibiting CYP3A-mediated metabolic activation and preserving GSH Homeostasis

Processing can reduce the total amount or active forms of toxic constituents entering the body by blocking the intestinal uptake of toxic precursors, inhibiting their hydrolysis and activation, or reducing their dissolution vehicles. Taking the Polygonaceae TCM *Polygoni Multiflori Radix* (Heshouwu) as an example, prolonged intake may induce varying degrees of adverse hepatic reactions, ranging from mild liver dysfunction to severe liver failure [168]. Its hepatotoxicity is closely associated with its toxic precursor, emodin-8-*O*-*β*-*D*-glucoside (EG). This constituent is absorbed via intestinal transporters (e.g., SGLT1 and GLUT2) and subsequently hydrolyzed to its aglycone, emodin (EMD), by β-glucosidases (e.g., LPH and CBG) within intestinal epithelial cells. The released EMD then accumulates in the liver, where its electrophilic nature enables covalent binding to the -SH of key cysteine residues in hepatic proteins, forming EMD-cysteine protein adducts, which ultimately trigger hepatocellular injury. Notably, processing with black soybean juice can suppress the activity of the aforementioned transporters and related hydrolases, thereby blocking the absorption, transport, and activation of the precursor toxin [169]. Consequently, the hepatotoxicity of *Polygoni Multiflori Radix* (Heshouwu) is significantly reduced [170] (Fig. 7A).

In addition, when *Armeniacae Semen Amarum* (Kuxingren) is decocted in water, moisture and appropriate temperature activate the endogenous enzyme system, causing rapid hydrolysis of amygdalin and releasing a large amount of highly toxic hydrogen cyanide (HCN) within a short period [171]. HCN inhibits cytochrome oxidase, leading to tissue hypoxia and, in severe cases, respiratory center paralysis and poisoning. A brief blanching treatment in boiling water thermally denatures and inactivates the enzyme proteins, while the relatively heat‑stable amygdalin is largely retained. After oral administration of the processed *Armeniacae Semen Amarum* (Kuxingren), due to the absence of enzymatic catalysis, amygdalin undergoes slow non‑enzymatic hydrolysis solely via gastric acid and intestinal microbiota, releasing trace amounts of HCN at a very low rate [172]. This process not only maintains an effective concentration of HCN for its antitussive and antiasthmatic effects but also keeps it far below the toxic threshold, thereby achieving the processing goal of toxicity reduction while preserving efficacy (Fig. 7B). Similarly, the processing of *Tribuli Fructus* (Jili) embodies the scientific principle of “enzyme inactivation for glycoside preservation”. Stir‑frying effectively inactivates the unique β‑glucosidase present in *Tribuli Fructus* (Jili), thereby blocking the conversion of furostanol saponins into the more toxic spirostanol saponins during storage at room temperature, and consequently reducing its hepatorenal toxicity [173].

In *Euphorbiae Semen* (Qianjinzi), the fatty oils serve as both carriers and absorption enhancers for toxic constituents. After defatting to produce frost, the oil content is significantly reduced, and consequently the dissolution and absorption of toxic constituents in the gastrointestinal tract are decreased, thus reducing its irritant toxicity [174]. All the above mechanisms illustrate that processing reduces toxicity from the outset by intervening in the critical steps through which toxic constituents enter the body.

#### Modulation of the pharmacokinetic behavior of toxic constituents

Processing can achieve toxicity reduction by modulating the pharmacokinetic behavior of toxic constituents, specifically through interventions in their absorption rate, tissue distribution, metabolic transformation, and elimination. For example, *Garcinia hanburyi* (Tenghuang), a toxic TCM of the Clusiaceae family, exhibits marked toxicity when administered orally. It readily irritates the gastrointestinal mucosa, increasing gastric acid secretion and intestinal peristalsis. High doses may induce severe abdominal pain, vomiting, and even intestinal hemorrhage; therefore, it must be steamed or boiled before clinical use to reduce its toxicity. Pharmacokinetic studies have shown that after processing, the toxic isomers R‑gambogic acid and S‑gambogic acid exhibit shortened T_max_, which contributes to reduced toxicity. In contrast, gambogenic acid—a constituent with stronger anticancer activity and lower toxicity—demonstrates significantly increased C_max_ and AUC_0−t_, thus maintaining or even enhancing the therapeutic effect while lowering toxicity [175].

Licorice juice, a widely used processing adjuvant, is employed in the processing of Aconitum species. Studies show that glycyrrhiza polysaccharides can modulate the pharmacokinetic profile of aconitine, promoting its rapid attainment of peak concentration and accelerating its metabolic clearance. This mechanism enables a swift onset of therapeutic action while preventing prolonged systemic accumulation and sustained high exposure to the toxic constituent. At the same time, glycyrrhiza polysaccharides enhance the bioavailability and prolong the duration of action of less toxic yet effective constituents such as benzoylmesaconine, collectively contributing to toxicity reduction and efficacy enhancement [176]. Furthermore, in accordance with the compatibility theories of “Xiangwei” or “Xiangsha”, processing with licorice juice can also alter the physical form of toxic constituents, specifically regulating their distribution and elimination in vivo. For instance, aconitine and proteins from licorice can self‑assemble via non‑covalent interactions into nano‑sized aggregates larger than 200 nm. Such aggregates cannot directly cross capillary walls to enter the bloodstream; instead, they are recognized and phagocytosed by macrophages, thereby blocking the distribution of aconitine to target organs such as the heart and significantly reducing its systemic toxicity [177].

#### Enhancement of systemic detoxification and metabolic capacity and restoration of metabolic homeostasis

*Euodiae Fructus* (Wuzhuyu), a Rutaceae TCM, is traditionally used to treat headaches, abdominal pain, epigastric distension, dysmenorrhea, and postpartum hemorrhage [178]. However, excessive use can lead to mild toxicity, which is markedly reduced after processing with licorice juice [179]. Research indicates that the hepatotoxicity of *Euodiae Fructus* (Wuzhuyu) is closely associated with its in vivo metabolic processes [180]. Its principal toxic constituent, evodiamine (EVO), undergoes cytochrome P450-mediated metabolic activation in the liver to generate highly electrophilic intermediates. These intermediates are typically conjugated with intracellular GSH, forming metabolites such as evodiamine-cysteinyl-glycine (EVO-Cys-Gly) and evodiamine-cysteine (EVO-Cys), which are subsequently eliminated via excretory pathways. However, when GSH is excessively depleted and cannot neutralize all reactive intermediates, the unconjugated electrophilic species can irreversibly bind to critical cysteine residues of functional hepatic proteins. This modification leads to protein inactivation or dysfunction, ultimately resulting in hepatocellular damage and hepatotoxicity. Interestingly, active constituents in licorice juice can alleviate the GSH depletion induced by *Euodiae Fructus* (Wuzhuyu), maintaining hepatic detoxification capacity. Additionally, by inhibiting CYP3A4 activity, they reduce toxic protein conjugation, thereby lessening the detoxification burden on the liver from the outset [181] (Fig. 7C). Systematic metabolomic studies further reveal that licorice juice not only directly inhibits specific toxicant-metabolizing enzymes but also acts as a “systemic modulator,” readjusting key metabolic networks—including primary bile acid biosynthesis, steroid hormone synthesis, and arachidonic acid metabolism—that are perturbed by the toxicity of *Euodiae Fructus* (Wuzhuyu) [182]. This restoration of hepatic metabolic homeostasis helps mitigate hepatocellular damage.

Similarly, *Rhizoma Dioscoreae Bulbiferae* (Huangyaozi), a Dioscoreaceae TCM used for conditions such as goiter and sore throat, is known to cause severe hepatotoxicity [183]. Its primary toxic constituent is the diterpenoid lactone diosbulbin B [184]. Research has shown that processing with licorice juice not only reduces the content of diosbulbin B but also, via glycyrrhizic acid present in the juice, inhibits CYP3A activity, thereby blocking the metabolic activation of diosbulbin B into highly reactive cis-enedial intermediates. This inhibition diminishes the conjugation of these intermediates with GSH, alleviating intracellular GSH depletion, and also prevents the aberrant covalent binding of the reactive metabolites to functional hepatic proteins, thereby reducing liver injury [185].

Likewise, the addition of licorice juice can mitigate the metabolic disturbances in amino acid, organic acid, and ketone metabolism induced by *Aconiti Radix Lateralis Praeparata* (Fuzi) in rats, suggesting that modulation of energy metabolism and ketone body pathways may be another important mechanism underlying the detoxifying effect of licorice juice processing exerts [186, 187]. Furthermore, processed *Strychni Semen* (Maqianzi) exhibits significantly reduced toxicity, while its anti‑inflammatory effects (reduction of paw swelling and levels of TNF‑α, IL‑1β, and IL‑6) remain essentially unchanged. Metabolomics studies have shown that processing markedly regulates pathways including phenylalanine metabolism, glycerophospholipid metabolism, the glutamate‑glutamine cycle, and arginine and proline metabolism, normalizing the levels of endogenous metabolites and thereby restoring metabolic homeostasis [188]. In summary, processing can enhance the clearance of toxic constituents and repair metabolic disorders by maintaining glutathione levels, regulating drug‑metabolizing enzyme activities, and modulating multiple endogenous metabolic pathways. Thus achieving the detoxification goal of enhancing systemic detoxification and metabolic capacity while restoring metabolic homeostasis.

## Conclusion

Based on the toxic TCMs recorded in the *Chinese Pharmacopoeia* (2025 Edition), this study systematically reviews their natural sources, chemical structural characteristics of the main toxic constituents, traditional processing detoxification methods, and underlying mechanisms. The results reveal that toxic TCMs derived from the same natural source category frequently contain identical or analogous types of toxic constituents, exhibit similar toxicological mechanisms, and are often processed using comparable strategies to promote chemical structural transformation of the toxic constituents for detoxification. These observations demonstrate a taxonomic commonality at the chemical transformation level, offering a new perspective for elucidating the universal principles underlying processing-induced detoxification. By integrating fragmentary research findings on individual TCMs, the detoxification mechanisms induced by processing can be categorized into three interrelated dimensions: physical detoxification (including physical removal of toxic constituents and alteration of the physical properties of decoction pieces), chemical transformation of toxic constituents, and modulation of in vivo processes.

Physical detoxification primarily relies on physical methods such as separation, purification, adjuvant adsorption, and thermal denaturation of proteins. Examples include removal of water-soluble toxic constituents through water processing, separation, adsorption of alkaloids using inert adjuvants, encapsulation of toxic needle crystals with Al(OH)_3_ colloids, and heat-induced denaturation of toxic proteins. These strategies all constitute regulatory approaches based on differences in physical properties. Chemical detoxification involves specific structural transformations of toxic constituents during processing. Due to differences in the chemical structures of their characteristic toxic constituents, toxic TCMs from various families exhibit distinct reaction pathways under heating or adjuvant treatment. These include hydrolysis and transesterification of alkaloids in Ranunculaceae species; isomerization and oxidation of alkaloids in Loganiaceae materials; hydrolysis of ester bonds and ring-opening of lactone or epoxide structures in diterpenoids of Euphorbiaceae plants; hydrolysis of glycosidic bonds in triterpenoid saponins of Phytolaccaceae herbs; formation of aluminum oxalate complexes in Araceae species; and alkaline-promoted hydrolytic ring-opening of cantharidin in Meloidae insects. These chemical reactions directly alter or disrupt toxic functional groups, thereby effectively reducing toxicity. Furthermore, fermentation‑based detoxification, as a distinct chemical detoxification strategy, utilizes microbial enzymatic systems to convert toxic constituents into less toxic products under mild conditions. Detoxification strategies based on in vivo processes primarily encompass three aspects. First, inhibiting intestinal absorption, hydrolytic activation, or reducing the dissolution carriers of toxic constituents decreases the total amount or active forms of toxic constituents entering the body at the source. Second, modulating the absorption rate, tissue distribution, metabolic transformation, and elimination of toxic constituents optimizes their pharmacokinetic behavior. Third, enhancing the body’s detoxification metabolic capacity and restoring metabolic homeostasis are achieved by maintaining glutathione levels, regulating drug‑metabolizing enzyme activities, and repairing the endogenous metabolic network. These strategies reduce toxicity while preserving or even enhancing therapeutic efficacy. It is noteworthy that the processing of TCMs is a continuous procedure in which physical operations such as heating and water treatment often simultaneously induce chemical changes, and these chemical changes directly affect the absorption, distribution, metabolism, and excretion of the drugs after administration. Therefore, these multi‑level detoxification mechanisms are not mutually independent but are closely interconnected and follow a sequential progression.

This review reveals a taxonomic commonality in detoxification based on chemical structure types, systematically categorizes the processing‑induced detoxification mechanisms into three interrelated dimensions (physical detoxification, chemical transformation of toxic constituents, and modulation of in vivo processes), and proposes an integrated research strategy of “physical detoxification – chemical transformation of toxic constituents – modulation of in vivo processes.” However, this review also has certain limitations. Although considerable progress has been made in the chemical and pharmacological research on many toxic TCMs, widely accepted detoxification processing methods have yet to be established. Current processing standards remain inconsistent, and systematic investigations into the principles underlying processing‑induced detoxification are still lacking. When reviewing the detoxification mechanisms, it was found that existing studies have primarily focused on changes in chemical composition, while research on alterations in the in vivo disposition of toxic constituents induced by processing remains relatively weak. Most studies remain at the level of phenomenological description, lacking in‑depth mechanistic analysis at the molecular level.

It is worth noting that improper processing not only fails to achieve detoxification but may even increase toxicity. For example, insufficient heating or inadequate duration can lead to incomplete transformation of toxic constituents, whereas excessive heating may generate new toxic degradation products. Therefore, establishing scientific and standardized processing procedures is essential for ensuring clinical safety. Based on the mechanistic analysis above, the optimization of modern processing techniques can be pursued from the following aspects. First, integrated control of multiple parameters during processing should be implemented, comprehensively considering the interactions among key parameters such as temperature, duration, adjuvant concentration, and pH, to establish multi-dimensional processing windows. Second, intelligent online monitoring technologies should be developed, employing sensing techniques such as near-infrared spectroscopy, electronic nose, and machine vision to capture real-time physicochemical characteristics of the TCMs during processing, and these data should be combined with machine learning algorithms to achieve synergistic multi-parameter control. Third, computational chemistry methods (e.g., density functional theory and molecular dynamics simulations) should be introduced to predict the reaction pathways, transition states, and toxicity of products derived from toxic constituents of different structural types under specific processing conditions, thereby guiding the selection of process parameters at the theoretical level.

Furthermore, fundamental differences exist between single-constituent detoxification and multi-constituent synergistic detoxification in the context of processing-induced detoxification strategies. Single-constituent detoxification is applicable to toxic TCMs that contain a single well-defined class of toxic constituents whose toxicity and efficacy can be dissociated. This approach primarily focuses on the chemical transformation or physical removal of a specific class of toxic constituents. The mechanism of this strategy is relatively straightforward, and the process is easy to control precisely. However, because the toxic constituents and the active (therapeutic) constituents are often identical or closely related, simply reducing the toxic constituents’ content may concurrently diminish therapeutic efficacy. Moreover, this strategy is often sensitive to processing conditions. In contrast, multi-constituent synergistic detoxification mainly relies on interactions among multiple constituents derived from processing adjuvants or the TCM itself to reduce toxicity through synergistic pathways, including physical, chemical, and metabolic mechanisms. This approach reduces toxicity while preserving, or even enhancing, the original therapeutic effects to the greatest extent possible. Nevertheless, this strategy involves complex mechanisms and poses significant challenges for quality control. It should also be noted that, in addition to processing-induced detoxification, the multi-constituent synergistic effects in TCM formula compatibility represent another important detoxification strategy. Through rational compatibility with other TCMs, the in vivo processes of toxic constituents can be modulated, or their toxic effects can be antagonized, thereby achieving toxicity reduction and efficacy enhancement. Therefore, in practical applications, the single-constituent or multi-constituent synergistic detoxification strategy should be selected based on the specific characteristics of the toxic TCMs, and the combined use of processing and formula compatibility deserves due attention.

Future research should prioritize toxic TCMs for which widely accepted processing methods for detoxification have not yet been established, develop systematic detoxification technologies and standardized processing protocols, and apply the aforementioned integrated research strategy. The strategy can be implemented as follows: First, observe the physical state changes and alterations in constituent dissolution caused by the processing procedure, while also considering physical interactions between adjuvants and toxic constituents (e.g., adsorption, encapsulation), to elucidate the detoxification effects at the physical level. On this basis, employ high‑resolution mass spectrometry techniques (LC‑MS/MS, GC‑MS, etc.) to dynamically track the structural evolution of toxicity‑related markers during processing, identify transformation products, and elucidate their reaction pathways, thereby clarifying the transformation mechanisms at the chemical level. Finally, validate the overall detoxification efficacy through in vivo pharmacokinetic and toxicological experiments to ensure the clinical safety of the processed products. In this process, particular emphasis should be placed on investigating processing‑induced changes in the in vivo behavior of toxic constituents. Given that in vivo processes are governed by complex regulatory networks involving multiple endogenous substances, systems biology approaches (e.g., metabolomics, proteomics) combined with artificial intelligence technologies should be introduced to achieve precise elucidation of processing-induced detoxification mechanisms and intelligent optimization of processing technologies.

## Data Availability

No datasets were generated or analysed during the current study.

## Abbreviations

AMPK: AMP‑activated protein kinase
AQP3: Aquaporin-3
AUC: Area under the curve
Bax: Bcl-2-associated X protein
Bcl-2: B‑cell lymphoma 2
CBG: Cytosolic broad‑specificity beta‑glucosidase
CHOP: C/EBP homologous protein
DART-MS: Direct analysis in real time mass spectrometry
DDAs: Diester-diterpenoid alkaloids
DDIT4: DNA damage inducible transcript 4
DESI-MSI: Desorption electrospray ionization mass spectrometry imaging
EG: Emodin‑8‑*O*‑*β*‑*D*‑glucoside
EMD: Emodin
EVO: Evodiamine
EVO-Cys: Evodiamine-cysteine conjugate
EVO-Cys-Gly: Evodiamine-cysteinyl-glycine conjugate
GLUT2: Glucose transporter 2
GSH: Glutathione
HCN: Hydrogen cyanide
HIF-1: Hypoxia‑inducible factor 1
IL-1β: Interleukin‑1beta
LPH: Lactase‑phlorizin hydrolase
MAPK: Mitogen‑activated protein kinase
MDA: Malondialdehyde
MDAs: Monoester-diterpenoid alkaloids
mTOR: Mammalian target of rapamycin
NDAs: Nonester‑diterpenoid alkaloids
NF-κB: Nuclear factor kappa‑light‑chain‑enhancer of activated B cells
NLRP3: NOD‑like receptor family pyrin domain containing 3
OATs: Organic anion transporters
PERK: PKR‑like ER kinase
PGC-1α: Peroxisome proliferator‑activated receptor gamma coactivator 1‑alpha
PKC: Protein kinase C
PMA: Phorbol 12‑myristate 13‑acetate
PPARγ: Peroxisome proliferator‑activated receptor gamma
RIP: Ribosome-inactivating protein
ROS: Reactive oxygen species
SGLT1: Sodium-glucose linked transporter 1
SOD: Superoxide dismutase
TCM: Traditional Chinese Medicine
TCMs: Traditional Chinese Medicines
TNF‑α: Tumor Necrosis Factor-alpha
